# The expansion of targetable biomarkers for CAR T cell therapy

**DOI:** 10.1186/s13046-018-0817-0

**Published:** 2018-07-21

**Authors:** Michelle H. Townsend, Gajendra Shrestha, Richard A. Robison, Kim L. O’Neill

**Affiliations:** 1 Department of Microbiology and Molecular Biology, Brigham Young University, 3142 LSB, Provo, UT 84602 USA; 2 Thunder Biotech, Highland, UT USA

**Keywords:** CAR T cell therapy, Biomarkers, Hematological malignancies, Solid tumors, Targets, Tumor associated antigens (TAA), Tumor specific antigens (TSA), Combination therapy

## Abstract

Biomarkers are an integral part of cancer management due to their use in risk assessment, screening, differential diagnosis, prognosis, prediction of response to treatment, and monitoring progress of disease. Recently, with the advent of Chimeric Antigen Receptor (CAR) T cell therapy, a new category of targetable biomarkers has emerged. These biomarkers are associated with the surface of malignant cells and serve as targets for directing cytotoxic T cells. The first biomarker target used for CAR T cell therapy was CD19, a B cell marker expressed highly on malignant B cells. With the success of CD19, the last decade has shown an explosion of new targetable biomarkers on a range of human malignancies. These surface targets have made it possible to provide directed, specific therapy that reduces healthy tissue destruction and preserves the patient’s immune system during treatment. As of May 2018, there are over 100 clinical trials underway that target over 25 different surface biomarkers in almost every human tissue. This expansion has led to not only promising results in terms of patient outcome, but has also led to an exponential growth in the investigation of new biomarkers that could potentially be utilized in CAR T cell therapy for treating patients. In this review, we discuss the biomarkers currently under investigation and point out several promising biomarkers in the preclinical stage of development that may be useful as targets.

## Background

As the new paradigm shift in cancer treatment, immunotherapy is the epitome of personalized medicine, as a patient’s immune system is enlisted to fight their own cancer. Originally manifest as monoclonal antibody therapy, immunotherapy now has a broadened definition that encompasses tumor vaccines, checkpoint blockades, bispecific antibodies, tumor infiltrating lymphocytes (TILs), and most recently, chimeric antigen receptor (CAR) T cell therapy. T cells are a critical component of the adaptive immune system as they not only orchestrate cytotoxic effects, but also provide long term cellular ‘memory’ of specific antigens [1]. Commonly, a patient will have TILs specific for their tumor but these cells are often retrained by the tumor microenvironment to become anergic and nonfunctional [2]. T cells endogenously require the interaction between MHC displayed peptides and their TCR to activate [3], but CAR T cells have been engineered to activate via a tumor-associated or tumor-specific antigen (TAA and TSA, respectively). CAR T cells are a “living drug” comprised of a targeting domain (single chain variable fragment (scFv), peptides, polypeptides, ligands, muteins, etc.) fused to the signaling domain of a T cell [4, 5]. Upon recognition and binding to the scFv target, the T cell activates and subsequent target cell killing is initiated. CAR T cell therapy has been revolutionary in the treatment of hematological malignancies with the targets CD19 and CD20 but has been unable to translate effectively to solid tumors. A major drawback for CAR therapy in solid malignancies is the lack of cancer-specific tumor targets. While hematological malignancies do not necessarily require complete antigen target specificity towards cancer cells, solid tumor targets are more delicate and targets ideally cannot be expressed on normal tissue. With the struggles facing CAR T cell therapy (on-target off-tumor cytotoxicity, persistence in vivo, immunosuppressive tumor microenvironment, cytokine release syndrome, etc.), biomarker discovery and specificity is essential for further CAR T cell development and success.

**Fig. 1:**
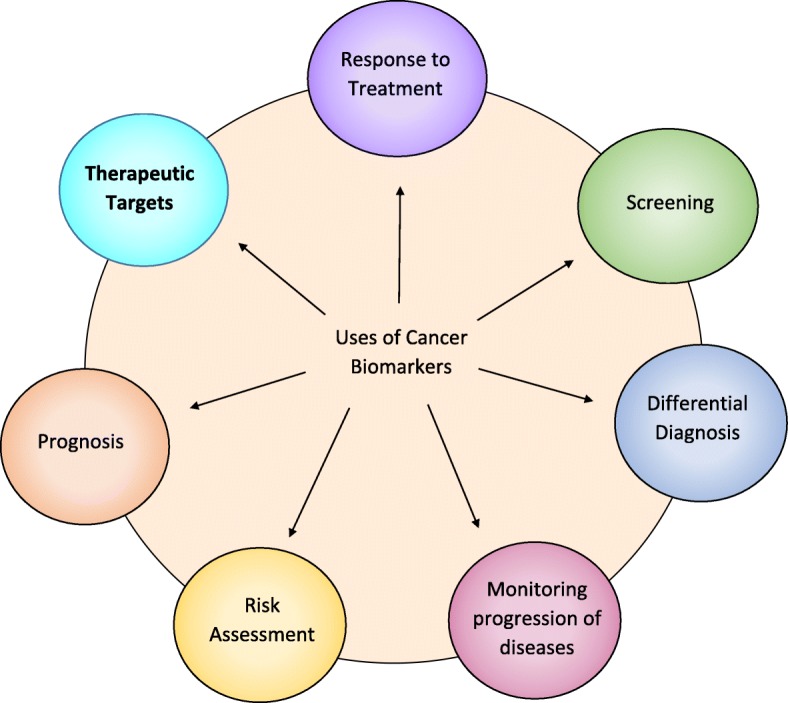
Uses of Cancer Biomarkers*.* Cancer biomarkers have had a historically proven useful for several different aspects of cancer patient care. With the advent of immunotherapy, surface cancer biomarkers are being utilized as therapeutic targets to direct and orchestrate an immune response in a cancer-specific fashion

**Fig. 2:**
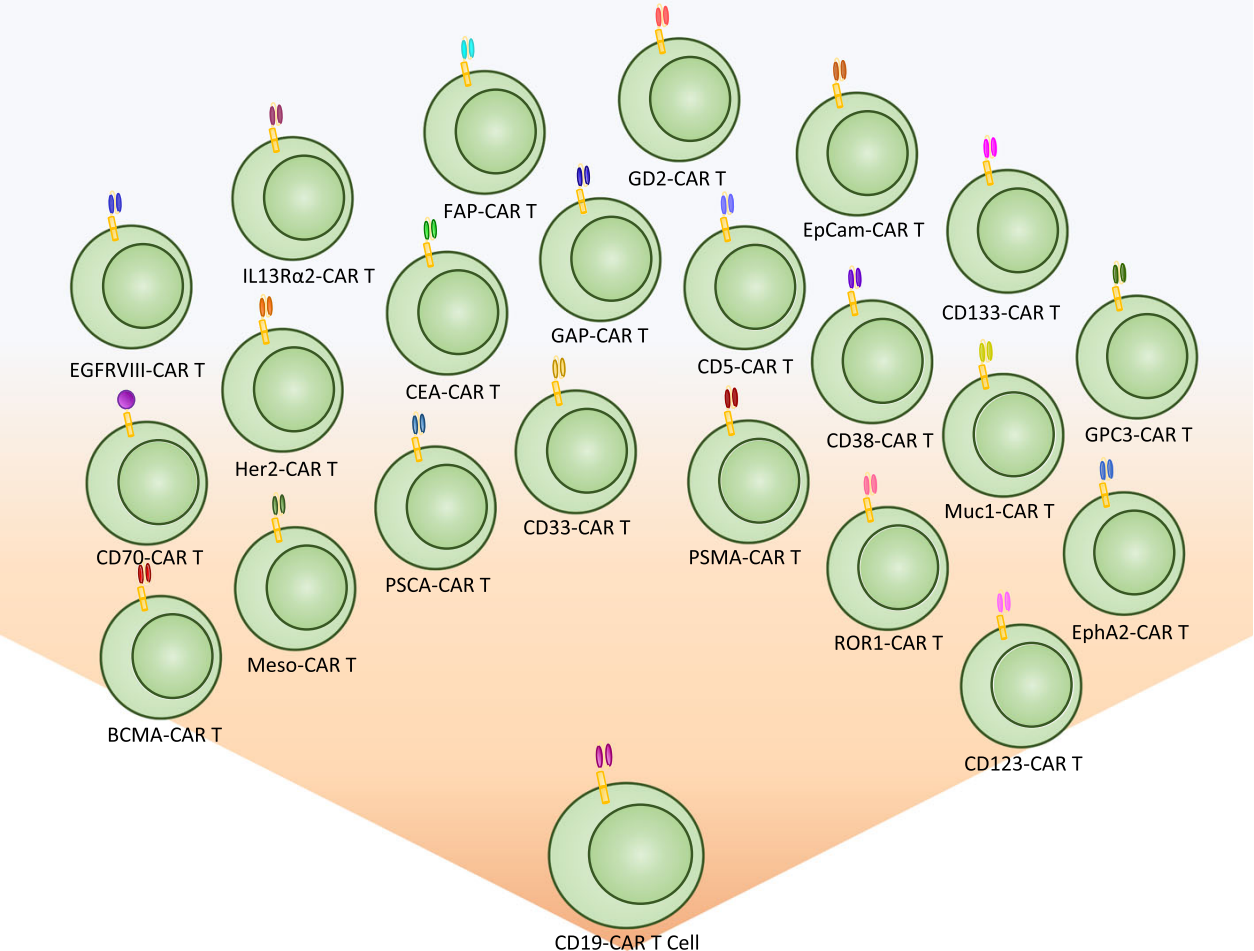
Current CAR T cells in clinical trials. From the initial success of CD-19 CAR T cell therapy, several new biomarker targets have emerged and are being tested in clinical trials. This expansion of targets has expanded CAR T cell therapy to the treatment of not just hematological malignancies, but also to solid tumors as well

With over 300 CAR T cell therapy clinical trials ongoing in CAR therapy as of May 2018, there has been an equally impressive effort to identify and characterize TAA or TSA surface biomarkers in solid tumors. Biomarkers have been an integral component of cancer for several decades, and with the expansion of CAR T cell therapy, a new category of therapeutic biomarkers has arisen. These markers can be used to direct CAR T cells to malignant target cells (Fig. 1). The effort to identify and characterize these therapeutic biomarkers has been substantial and has increased exponentially over the last decade. As a result, 18 surface biomarkers are currently being evaluated in clinical trials (Fig. 2). In addition, there is also a significant number of pre-clinical biomarkers that have shown promise as targets for CAR therapy due to their unique expression on cancer cells. Here, we summarize the biomarkers currently under investigation in clinical trials for both hematological and solid malignancies, along with those that may prove useful in future CAR therapies for solid tumors.

## Surface biomarkers have expanded significantly over the last decade

**Fig. 3:**
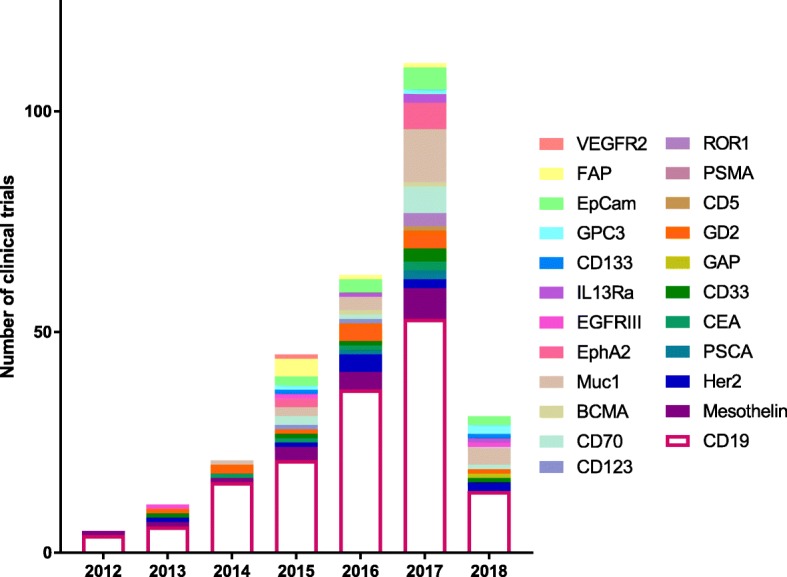
Clinical trial Biomarkers as of May 2018 by year. The expansion of CAR targets is shown as the diversity and number of clinical trials has exponentially increased from 2012. Not only are there more clinical trials utilizing CAR T cell therapy, there are also more targets being evaluated

CAR T cell therapy was initially conceptualized in 1989 [6] and was recognized as an effective therapeutic after targeting CD19 for the treatment of lymphomas and leukemias [7–9]. This led to an exponential growth in CAR therapy and as a direct consequence, in surface biomarker discovery (Fig. 3). In 2012, there were a total of 5 clinical trials, four targeting CD19 and one targeting Mesothelin. This number has continued to grow and the number of biomarkers tested in a clinical setting has also expanded from 2 to 25. The year 2017 saw more clinical trials than any previous year with 111 initiated, targeting 17 different biomarkers (Table 1). This growth demonstrates not only the efficacy of CAR T cell therapy, but also the huge push in immunotherapy to find new and better targets.

**Table 1:** Current Clinical Trials (as of April 2018)

Target	Name	Function	Disease	Clinical Trials in 2018
CD19	Cluster of Differentiation 19	Dominant signaling component on mature B cells	ALL, B cell lymphoma, leukemia, Non-Hodgkin lymphoma,	NCT03366350^b^, NCT03366324^b^, NCT02546739^b^, NCT03448393^b^, NCT03467256^b^, NCT03488160^b^, NCT03016377^b^, NCT03468153^b^, NCT03483688^b^, NCT03398967^b^, NCT03229876^b^, NCT03455972^b^, NCT03423706^b^, NCT03497533^b^
Mesothelin		exact function of mesothelin in these normal mesothelial cells is unclear.	Pancreatic cancer, Cervical Cancer, Ovarian Cancer, Lung Cancer, Peritoneal carcinoma, Fallopian tube cancer, Colorectal Cancer, Breast Cancer	NCT02930993^a^, NCT03182803^a^, NCT03030001^a^, NCT02706782^a^, NCT01583686^a^, NCT03356795^a^, NCT03054298^a^, NCT03267173^a^, NCT02792114^a^, NCT02959151^a^, NCT02580747^a^, NCT02414269^a^, NCT02465983^a^, NCT03323944^a^,
Her2	Human Epidermal Growth Factor Receptor 2	Activate intracellular signaling pathways in response to extracellular signals.	CNS tumor, Breast Cancer, Ovarian Cancer, Lung Cancer, Gastric Cancer, Colorectal Cancer, Glioma, Pancreatic Cancer, Glioblastoma	NCT03500991^b^, NCT03423992^b^, NCT02713984^a^, NCT03267173^a^, NCT02792114^a^, NCT02442297^a^, NCT00889954^a^, NCT03423992^a^, NCT01109095^a^, NCT02706392^a^, NCT00902044^a^, NCT03389230^a^, NCT01818323^a^
PSCA	Prostate Stem Cell Antigen	Not well understood	Pancreatic cancer, lung cancer	CT03198052^a^, NCT02744287^a^, NCT03267173^a^
CEA	Carcinoembryonic antigen	Cell adhesion	Liver metastases, lung cancer, colorectal cancer, gastric cancer, breast cancer, pancreatic cancer,	NCT02850536^a^, NCT02349724^a^, NCT03267173^a^, NCT02959151_,_ NCT01212887^a^
CD33	Siglec-3	Transmembrane receptor on myeloid lineage	Myeloid leukemia,	NCT03473457^b^, NCT02958397^a^, NCT03126864^a^, NCT03222674^a^,
GAP	GTPase-activating protein	Terminating G protein signaling	Solid tumors	NCT02932956^b^
GD2	Ganglioside G2		Glioma, Cervical cancer, sarcoma, neuroblastoma,	NCT03423992^b^, NCT03356795^a^, NCT02992210^a^, NCT01953900^a^, NCT02761915, NCT03373097^a^, NCT02765243^a^, NCT03423992^a^, NCT03294954^a^, NCT03356782^a^, NCT02919046^a^,
CD5	Cluster of differentiation 5	TCR inhibitory molecule	T cell acute lymphoblastic lymphoma, T-non-Hodgkin lymphoma,	NCT03081910^a^,
PSMA (PSMA/TGFβ)	Prostate specific membrane antigen	Transmembrane protein	Cervical cancer, Prostate cancer, Bladder cancer	NCT03356795^a^, NCT03089203^a^ (-TGFβ), NCT03185468^a^, NCT01140373^a^
ROR1	Receptor Tyrosine Kinase like Orphan Receptor 1	Modulates neurite growth in the CNS	Breast cancer, lung cancer, lymphoblastic leukemia,	NCT02706392^a^,
CD123	IL-3RA	Involved in hematopoietic progenitor cell differentiation and proliferation	AML, Leukemia,	NCT03473457^b^, NCT03125577^a^, NCT02937103^a^, NCT03114670^a^, NCT02159495^a^, NCT03098355^a^, NCT03222674^a^, NCT03203369^a^, NCT03190278^a^,
CD70	Cluster of differentiation 70	Induces proliferation of costimulated T cells	B cell malignancies, pancreatic cancer, renal cell cancer, breast cancer, melanoma, ovarian cancer	NCT03125577^a^, NCT02830724^a^,
CD38	Cluster of differentiation 38	Cell adhesion, signal transduction, and calcium signaling	Myeloma,	NCT03464916^b^, NCT03473496^b^, NCT03473457^b^, NCT03125577^a^, NCT03222674^a^, NCT03271632^a^,
BCMA	B cell maturation antigen	Mediates the survival of plasma cells	Myeloma	NCT03448978^b^, NCT03473496^b^, NCT03430011^b^, NCT03455972^b^, NCT02954445^a^, NCT03322735^a^, NCT03338972^a^, NCT03318861^a^, NCT02215967^a^, NCT03093168^a^, NCT03274219^a^, NCT03302403^a^, NCT03492268^a^, NCT03288493^a^, NCT03070327^a^, NCT03196414^a^, NCT03448978^a^, NCT02958410^a^, NCT03287804^a^, NCT03473496^a^, NCT03380039^a^, NCT03430011^a^, NCT03361748^a^, NCT03455972^a^, NCT02546167^a^, NCT03271632^a^
Muc1	Mucin 1	Mucous barrier formation on epithelial surfaces	Sarcoma, Leukemia, Pancreatic cancer, cervical cancer, lung cancer, hepatocellular carcinoma, breast cancer, glioma, colorectal cancer, gastric cancer	NCT03179007^a^, NCT02587689^a^, NCT02617134^a^, NCT03198052^a^, NCT03356795^a^, NCT03267173^a^, NCT03222674^a^, NCT03356782^a^
EphA2	Ephrin type-A receptor 2 precursor	Eph-ephrin bidirectional signaling pathway of mammalian cells	Glioma	NCT03423992^b^
EGFRVIII	Epidermal growth factor receptor variant III	Cell differentiation and proliferation	Glioblastoma	NCT03283631^b^
IL13Ra2	Interleukin 13 receptor, alpha 2	Signal processing via Jak-STAT	Glioma	NCT02208362^a^
CD133	Prominin-1	unknown	Glioma, AML, Liver Cancer, Pancreatic Cancer, Ovarian Tumor, Colorectal Cancer, Breast Cancer	NCT03473457^b^, NCT03356782^a^, NCT03423992^b^
GPC3	Glypican 3	Regulate cell growth, division, and survival	Heptocellular carcinoma, lung cancer, Lymphoma, Leukemia, Pancreatic Cancer, Colorectal Cancer	NCT02905188^b^, NCT02932956^b^, NCT02715362^a^, NCT03130712^a^, NCT02395250^a^, NCT02876978^a^, NCT03198546^a^, NCT02723942^a^, NCT03084380^a^, NCT03302403^a^, NCT03146234^a^, NCT02959151^a^,
EpCam	Epithelial cell adhesion molecule precursor	Embryonic stem cell proliferation and differentiation	Breast Cancer, Colon Cancer, Pancreatic Cancer, Esophageal Carcinoma, Gastric Cancer, Prostate Cancer, Hepatic Carcinoma, Lymphoma, Leukemia	NCT02915445^a^, NCT03013712^a^, NCT02729493^a^, NCT02725125^a^, NCT02728882^a^, NCT02735291^a^
FAP	Fibroblast activation protein alpha	Neuropeptide regulation. hFGF21 inactivation	Pleural Mesothelioma	NCT01722149^a^

Note. ^a^; indicate trials ongoing/active, ^b^; indicate trials that started in 2018

## Current clinical targets for hematological malignancies

**Fig. 4:**
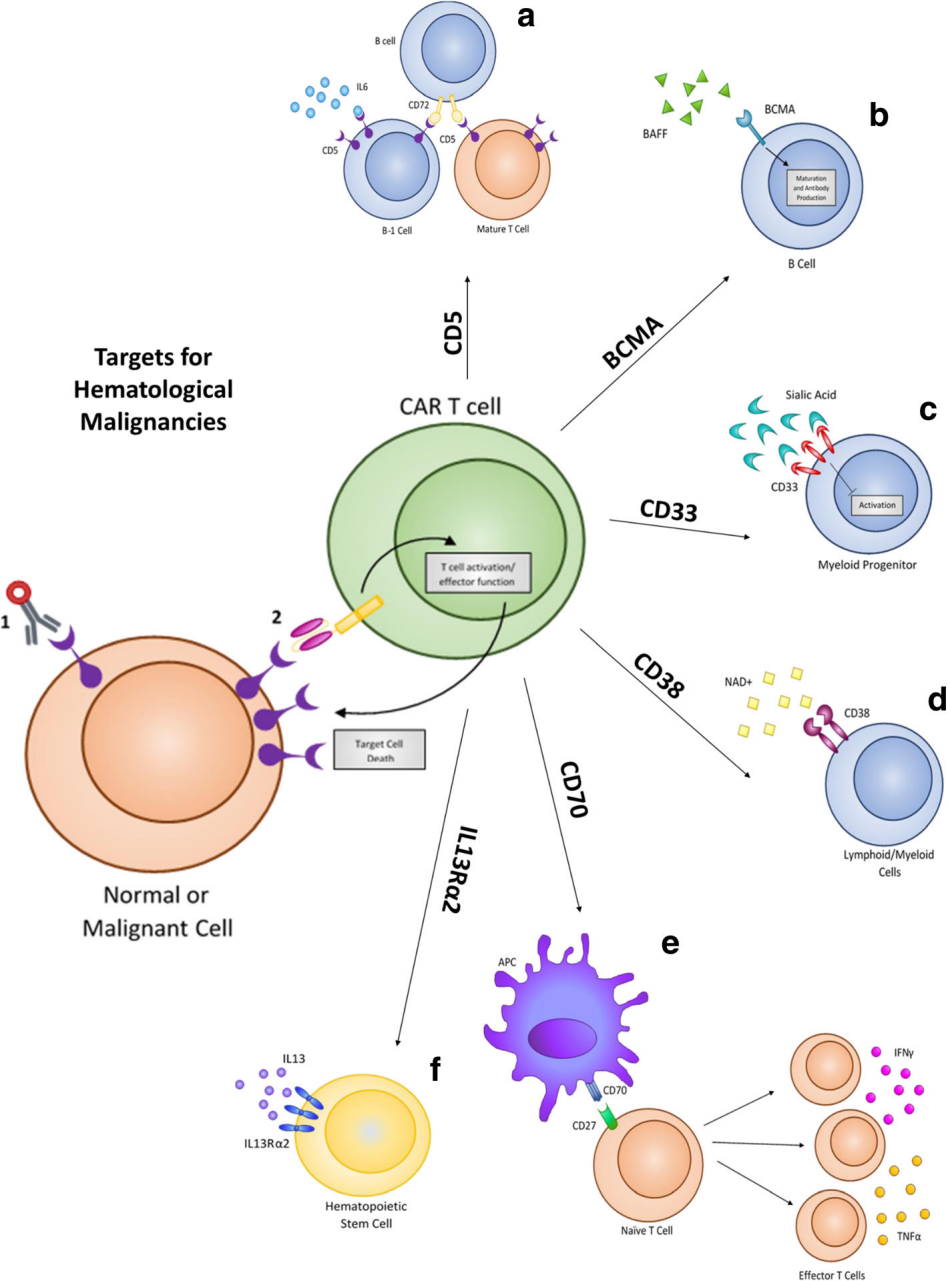
Biomarker targets for hematological malignancies. The endogenous function of each of **a** CD5, **b** BCMA, **c** CD33, **d** CD38, **e** CD70, and **f** IL13Rα2 are shown. These targets are all being utilized to treat hematological malignancies in clinical trials. They are not cancer-specific and do have expression on normal cells, but have an elevation within cancer that is being used for targeting

As the most studied and researched target for CAR therapy, CD19 has shown impressive success in clinical settings to treat Acute Lymphoblastic Leukemia (ALL), Non-Hodgkin Lymphoma (NHL), and Chronic Lymphocytic Leukemia (CLL) [10]. Despite the high levels of complete response rates in patients, relapse from CD19 CAR therapy can occur via a suppressive tumor microenvironment or antigen escape [11–13]. With this in mind, new targets are being identified and evaluated to treat hematological malignancies. Among these new targets are CD5, CD123, CD33, CD70, CD38, and BCMA. These same targets have already shown promise using drug-conjugated antibodies, and several have been FDA approved for treatment (Figs. 1, 2, 3 and 4). These biomarkers are now being evaluated as targets for adoptive T cell CAR therapy to treat hematological malignancies.

### CD5

CD5 is a negative regulator of TCR signaling and is expressed on the surface of most T cells and on a specific subpopulation of B cells (B-1) found most commonly in fetal cells [14] (Fig. 4a). CD5 has high expression in approximately 80% of T-cell acute lymphoblastic leukemia (T-ALL) and T cell lymphomas along and also has significant expression on B-cell lymphomas [15]. CD5 was first utilized as an immunotherapy treatment via immunotoxin-conjugated antibodies [16–22] that aided in the depletion of malignant T cell populations in treated patients. More recently, CD5 has been utilized as a CAR target to treat T cell malignancies directly. As CD5 is not cancer specific, this treatment results in T cell aplasia [23, 24]. While this therapy is effective in eliminating malignant T cells, sustained T cell aplasia is a potentially undesirable outcome of treatment.

### IL3Rα

Interleukin-3 receptor alpha chain (IL3Rα or CD123) is a surface receptor found overexpressed in several hematological malignancies including blastic plasmacytoid dendritic cell neoplasm (BPDCN) [25], hairy cell leukemia [26, 27], B-cell acute lymphocytic leukemia (B-ALL) [26, 28], and Acute myeloblastic leukemia (AML) [29, 30]. As the receptor expression is limited on hematopoietic stem cells, the receptor has promising use as a targetable biomarker for CAR therapy [30, 31] (Fig. 4f). Initial targeting of IL3Rα was conducted utilizing the natural ligand, IL-3, but CAR T cell approaches are now being utilized to further target this receptor to treat primarily AML patients. Initial trials with CD123 CAR cells showed potent cytotoxicity against AML cells within mice [32–35] and in human patients [36]. This preliminary success has led to its further testing in clinical trials, evaluating this therapy for both safety and efficacy against AML. IL3Rα, like CD5, is not cancer specific, and the consequence of CD5 CAR T cells is severe myeloablation [37, 38].

### CD33

CD33 is a transmembrane receptor that binds sialic acid and causes inhibition of activation. The protein is expressed on AML blasts and normal myeloid progenitors [39–43] (Fig. 4c). Because CD33 is absent in adult pluripotent hematopoietic stem cells and has elevated expression on approximately 85–90% of AML patients, the antigen has gained clinical significance as a TAA [44–46]. In initial trials testing the efficacy of CD33 CAR T cells, patients showed signs of an inflammatory reaction in response to infused CAR T cells: chills, fever, and elevated cytokine levels. This resulted in reduced blasts within the bone marrow following two weeks of therapy [47]. Following these preliminary tests, clinical trials are ongoing to determine if CD33 is a safe and effective treatment for myeloid leukemia.

### CD70

CD70 is a target that is being utilized to treat both hematological malignancies as well as solid tumors (Table 1). CD70 is the membrane-bound ligand of the CD27 receptor (TNF superfamily) [48–50] (Fig. 4e). Expression of CD70 is limited to diffuse large B-cell and follicular lymphomas, as well as Hodgkin’s lymphoma, multiple myeloma, and EBV-associated malignancies [51–55]. Additionally, CD70 is also expressed on other malignancies such as glioma [56–59], breast cancer [60, 61], renal cell carcinoma [51, 62–64], ovarian cancer [65–67], and pancreatic cancer [65, 68]. Targeting this antigen is feasible as CD70/CD27 signaling is not essential for the development of a functional immune system as CD27^−/−^ mice recover from infection in a similar time frame as CD27^WT^ mice [69, 70]. Targeting was first performed using monoclonal antibodies against CD70, and this showed promise in animal models [51, 71, 72]. CD70 CAR T cells contain the human CD27, the natural binding partner of CD70, fused to the CAR signaling domain [48].

### CD38

CD38 is a glycoprotein associated within lipid rafts and is specific to cell surface receptors that function to regulate calcium flux and mediate signal transduction in both lymphoid and myeloid cells [73–75]. While CD38 is expressed consistently on myeloma cells [73, 76], it’s expression is limited on normal lymphoid and myeloid cells [77] (Fig. 4d). As a TAA, CD38 has been used as a target via monoclonal antibody treatment (Daratumumab) [73], which was approved by the FDA in 2015 for patients with multiple myeloma [78]. Daratumumab showed an overall response rate of 31%, which demonstrates the success of utilizing CD38 as a target. CD38 CAR T cells have shown similar efficacy against double-hit lymphoma cells (MYC rearrangement along with BCL2 or BCL6 rearrangement) [79]. With promising data, CD38 CAR T cells are currently in phase I trials against myeloma to test safety and dosing.

### BCMA

B cell maturation antigen (BCMA) is a TNF receptor that binds B-cell activating factor (BAFF) and is universally expressed on myeloma cells but has insignificant expression on major adult organs [80] (Fig. 4b). BCMA is exclusively expressed in B-cell lineage cells, and is expressed during plasma cell differentiation [81]. In preclinical models, anti-BCMA CAR T cells have shown effective killing of myeloma cells both in vitro and in vivo [82, 83]. Following Phase I safety studies, some patients experienced neurotoxicity and cytokine release syndrome, which are common side effects of CAR T cell treatment [84]. Other side effects of targeting BCMA are similar to those of other hematological malignancies, as patients suffer from partial or complete B cell aplasia.

## Current clinical targets for solid tumors

**Fig. 5:**
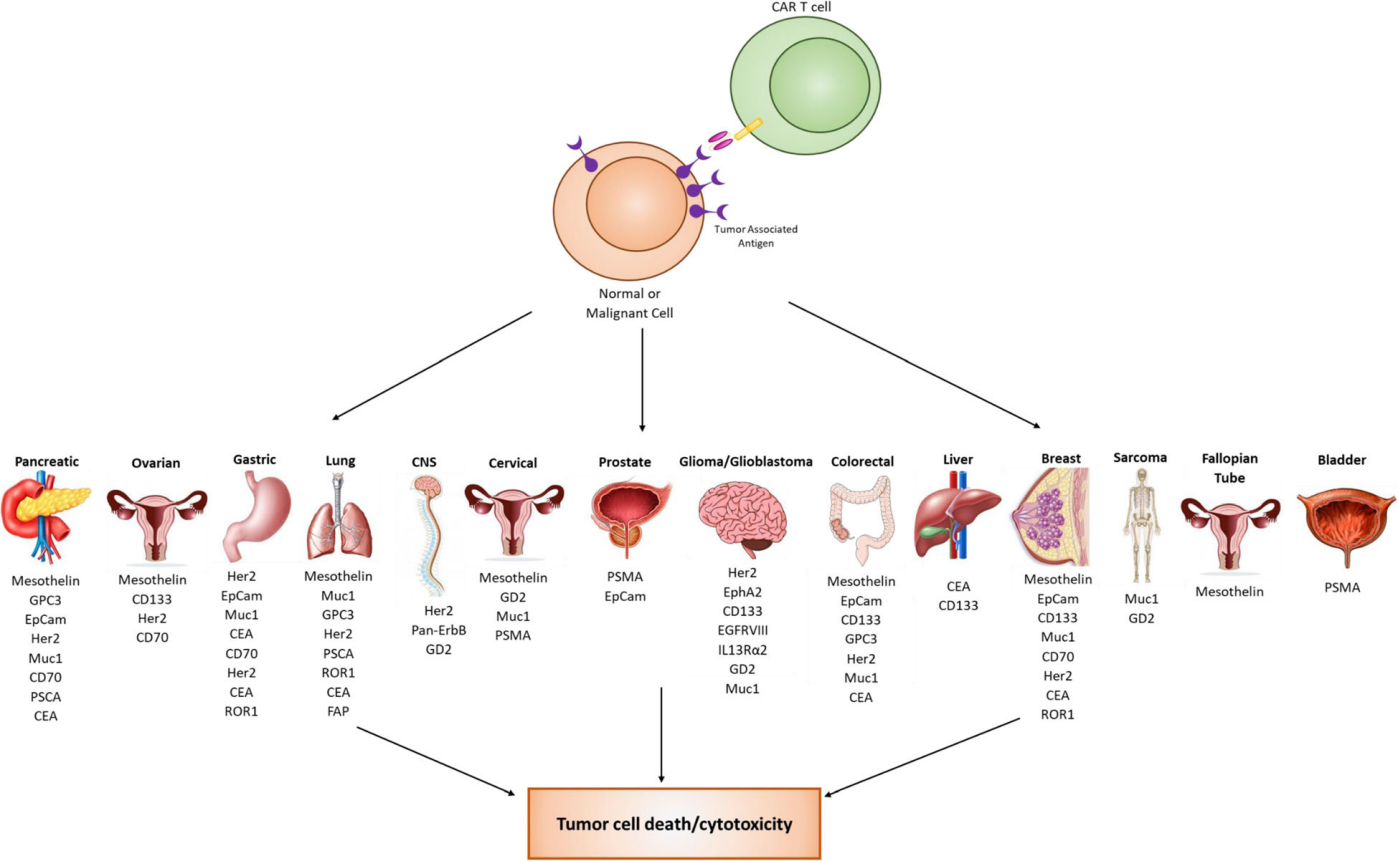
Biomarker targets for solid malignancies. Over 14 different organ types are currently being targeted using a variety of different biomarkers. Many biomarker targets have expression in several different cancer types

While CAR T cell therapy has been very successful against hematological malignancies, it has been challenging to apply this technology to solid tumors. This challenge has resulted in a strong effort to discover biomarkers for solid malignancies. As such, there are 17 biomarkers currently in clinical trials for solid tumors (Fig. 5).

### Mesothelin

Mesothelin (MSLN), the second most frequently targeted biomarker after CD19, has emerged as an attractive target for cancer immunotherapy. MSLN is a cell-surface glycoprotein with presence in the sera of cancer patients as soluble MSLN-related peptide (SMRP). Within normal tissue, the expression of MSLN is restricted to mesothelial cells lining the pericardium, peritoneum, and pleura. Yet, in cancer cells, MSLN is overexpressed on nearly a third of human malignancies [85]. Elevated levels of MSLN have been reported on ovarian cancers [86, 87], non-small-cell lung cancers [88, 89], breast cancers [90, 91], esophageal cancers [92], colon and gastric cancers [93], and pancreatic cancers [94]. In addition, Lamberts et al. reported MSLN expression in other solid tumors such as thyroid cancer, renal cancer, and synovial sarcoma [95]. The biological function of MSLN is nonessential given that MSLN^−/−^ mice do not show any phenotypic abnormalities [96]. However, the overexpression of MSLN has been associated with cancer cell proliferation, increased local invasion and metastasis, and resistance to apoptosis induced by cytotoxic agents [91, 97–99]. MSLN-CAR T cells have been created and tested against ovarian cancer, and lung cancer [97]. These CAR T cells have shown significant increases in T cell proliferation, T cell redistribution to metastatic sites, reduction in tumor burden, and increased overall survival. This promising pre-clinical data has led to several Phase I clinical trials to test the safety and efficacy of MSLN CAR T cell therapy against several tumors. Initial Phase I clinical trials have shown transient expression of the MSLN-CAR T cells and minimal cytokine release syndrome or on-target, off-tumor effects (NCT01355965, NCTO 02159716 & NCTO01897415). A single infusion of MSLN-CAR T cells resulted in decreased tumor burden and patients had no signs of long-term toxicities 1–2 months post infusion [100].

### Her2

HER2 (Human epidermal growth factor 2) is a transmembrane tyrosine kinase in the ERBB family. The HER2 receptor plays an important role in normal cell growth and differentiation, activating PI3K/Akt and RAS/Raf/MEK/MAPK pathways [101]. Studies have reported HER2 protein overexpression, gene amplification, and mutation in many cancers including breast, lung, colorectal, brain, ovarian, and pancreas [102]. Overexpression of HER2 has been found to be associated with increased tumor cell proliferation and invasion [103], decreased response to hormonal treatment [104], and resistance to apoptosis [105]. HER2 has been targeted utilizing DNA vaccines, peptide vaccines, and dendritic vaccines which have shown promising results in both preclinical and early clinical studies [106, 107]. Trastuzumab, a humanized monoclonal antibody developed to target overexpressed HER2 receptor, has also shown success as an immunotherapy treatment. Trastuzumab, along with chemotherapy, has increased overall survival and risk of recurrence compared to chemotherapy alone in HER2 overexpressing breast cancer patients [108]. Several groups have reported the anti-tumor activity, persistence, and application feasibility of HER2 CAR T cells preclinically in HER2 overexpressing cancer as an alternative targeted therapy [109–111]. The success of preclinical experiments of HER2 CAR T cell has led to the initiation of several clinical trials for the treatment of various cancers [112–114]. Additionally, Her2 is also used as a target in combinatorial therapy engaging multiple targets as well as modified receptors that enhance T cell signaling. T1E28z CAR T cells engage multiple ErbB dimers, including Her2-containing heterodimers. The CAR is co-expressed with a chimeric cytokine receptor called 4αβ that amplifies mitogenic stimulus delivered by IL-4, providing a convenient tool to enrich CAR T cells ex vivo [115]. Initial trials using these combinatorial CARs have shown safe intra-tumoral administration in patients with advanced head and neck squamous cancer [116].

### GD2

GD2 is a ganglioside antigen that is expressed on the surface of several malignancies including neuroblastoma [117], glioma, cervical cancer, and sarcoma [118, 119]. The normal expression of the protein is limited to neurons, melanocytes, and peripheral nerve fibers [119–121]. One of the most successful trial reports for CARs in solid tumors has been using GD2 as a target for neuroblastoma [122–125]. Not only did GD-2 CAR T cells induce a response in 30% of patients, including a complete remission in 3 patients, but researchers found long term persistence of the CAR T cells post treatment, which subsequently reduced tumor recurrence/progression [125]. Meanwhile, GD2 monoclonal antibodies (Dinutuximab) have been effective for the control of neuroblastoma [119, 126–128] and this product is currently FDA approved for that application. There have been some observed cytotoxicities associated with targeting GD2, such as sensorimotor demyelinating polyneuropathy presumably caused by on-target toxicity affected myelinated peripheral nerve fibers [120]. In preclinical models, severe lethal CNS toxicity caused by CAR T cell infiltration and proliferation within the brain resulted in neuronal destruction [129]. Therefore, although there has been success utilizing CAR therapy in patients, necessary precautions need to be taken to avoid neurotoxicity as GD2 has expression in normal neural cells. GD2, as of May 2018, has 10 ongoing clinical CAR T cell trials targeting primarily neuroblastoma. A majority of these clinical trials are in phase I status to determine the safety of the treatment. One of the clinical trials (NCT02765243) is testing the incorporation of a kill switch, which is an engineered suicide gene (iCasp9) to help avoid neurotoxicity.

### MUC1

MUC1 is a large transmembrane glycoprotein that is transcriptionally upregulated in breast and ovarian tumors [130, 131]. MUC1 expression is confined to normal luminal epithelium, and the expression is lost upon transformation [132–136]. MUC1 has recently become an interesting target in cancer immunotherapy because of the overexpression of aberrantly glycosylated MUC1 in most solid tumors and several hematological malignancies. This is in addition to the role of MUC1 in cancer progression, invasion, metastasis, angiogenesis, and chemoresistance. Although expressed significantly on malignant cells, MUC1 targeting presents some complications as MUC1 is shed and may inhibit tumor antibody binding/recognition [137]. MUC1 also has the ability to inhibit T cell function and thereby promotes an anti-inflammatory TME [138]. CAR T-cell therapy targeting MUC1 has been beset with several challenges such as steric hindrance and glycosylation-related epitope heterogeneity [139]. Following CAR optimization with tripartite endodomains and high affinity screening for effective ScFv fragments, MUC1-CAR T cells showed significant delays in tumor growth in mouse xenograft models [139]. MUC1-CAR T cells also show enhanced proliferation, increased IFN-ϒ secretion, and enhanced anti-tumor efficacy when compared to control CAR T cells in vitro [140]. Based on the success of these preclinical MUC1-CAR T cells, several clinical trials targeting MUC1 in several cancer types have begun. Early phase 1 clinical trials revealed no initial adverse side-effects and patient cytokine levels increased, indicating a positive response as tumor necrosis was observed [141].

### GPC3

Glypican-3 (GPC3) is a GPI bound sulfate proteoglycan involved in cellular growth, differentiation, and migration [142, 143]. GPC3 shows elevated expression in approximately 75% of hepatocellular carcinoma samples, but had no expression in corresponding normal tissue [144, 145]. GPC3 is also elevated within breast cancer [146], melanoma [147], and pancreatic cancer [148, 149] demonstrating its use across a wide variety of cancer types. GPC3 CAR T cells showed promising preclinical results targeting tumors in mouse xenograft models [150]. In human trials there was minimal toxicity and all patients tolerated the treatment (NCT02395250) [151]. Further clinical trials targeting lung cancer, pancreatic cancer, and colorectal cancer are ongoing.

### IL13Rα2

There are currently two clinical trials, one initiated in 2015 and one in 2018, testing the efficacy and safety of IL13Rα2 directed CAR T cells against glioma patients. IL-13 is a T helper 2 (TH2) derived cytokine involved in immune regulation. IL13Rα2 is an IL-13 receptor that acts as a decoy by directly competing with the IL13Rα1 receptor to elicit downstream STAT signaling [152, 153]. IL13Rα2 receptors are upregulated in approximately 50% of glioma patients and have a strong correlation with poor survival [154]. As a gene that is highly expressed in tumor infiltrating macrophages (TIM) and tumor-associated macrophages (TAM), but shows minimal expression in normal brain tissue, IL13Rα2 has been previously studied as a cancer vaccine, and more recently as a direct target for CAR therapy. Initially, IL13Rα2 CAR T cells were developed utilizing a membrane-tethered IL13 ligand mutated at residue 13 (E➔Y) [154] as the antigen recognition domain. Unfortunately, it was determined that these domains also recognized IL13Rα1 receptors as well, which raised significant safety concerns. New CAR T cell constructs targeting IL13Rα2 therapy rely on scFv-based targeting. With this modification in antigen specificity, scFv-based IL13Rα2 CARs induce tumor regression in mouse xenograft models of glioma and show insignificant recognition of IL13Rα1 receptors [155]. In 2016, a patient who received Il13Rα2 CAR T cells through two intracranial delivery routes followed by infusions into the ventricular system over 220 days showed regression of all intracranial and spinal tumors which continued 7.5 months after the initiation of the therapy [156]. This remarkable sustained response by this patient demonstrates the promise of targeting IL13Rα2.

### PSCA

Prostate stem cell antigen (PSCA) is a serine protease [157, 158] expressed in the basal cells of normal prostate cells [159] and is overexpressed in approximately 80% of prostate cancers [160–163]. In addition, PSCA expression increases with both high Gleason score, and metastasis [162]. The expression of PSCA is limited to the basal cell epithelium in the prostatic epithelium [160]. As a protein attached to the cell surface via a GPI-anchor, it serves as an ideal target for prostate cancer and further metastatic sites [162]. PSCA has also been found expressed on other cancer types such as gastric cancer, gallbladder adenocarcinoma [164–166], non-small-cell lung cancer [159, 167], ad pancreatic cancer [168]. In humanized mouse models, CAR T cells targeting PSCA induced significant antitumor activity in pancreatic cancer [168]. Although initial results have been promising, preclinical reports have shown that tumors can escape PSCA-CAR T cells and while treatment does prolong survival, it does not necessarily eradicate PSCA-expressing tumors [169, 170].

### VEGFR2

Vascular endothelial growth factor receptor 2 (VEGFR2) is an important mediator of tumor angiogenesis [171, 172]. VEGFR2 is involved in microvascular permeability, endothelial cell proliferation, invasion, migration, and survival [173]. Overexpression of VEGFR2 has been associated with increased metastasis in several malignancies [174, 175], and VEGFR2 expression has also been shown on squamous cell carcinomas of the head and neck [176], colorectal cancer [177, 178], breast cancer [179, 180], and NSCLC [181–183]. While overexpressed in cancer, the expression of VEGFR2 in normal tissue is restricted to endothelia and mesothelial [184]. Initial targeting of VEGFR2 with monoclonal antibodies has resulted in growth inhibition and decreased micro vessel density while simultaneously inducing tumor cell apoptosis and necrosis [185, 186]. These preclinical results have been shown in NSCLC, renal carcinoma, hepatocellular carcinoma, melanoma, ovarian cancer, and colorectal cancer [174, 187–191]. To date, only one clinical trial has been enrolled utilizing CAR T cells against VEGFR2 (NCT01218867) [192].

### CEA

Carcinoembryonic antigen (CEA) is a glycoprotein on the surface of several carcinomas [193]. The most studied use for CEA as a surface biomarker has been in liver metastasis, especially originating from colorectal cancer [194–196]. CEA is also significantly expressed on the surface of gastric cancer, pancreatic cancer, ovarian cancer, and lung cancers [197]. While CEA is expressed on the surface of some normal cells, including epithelial cells in the pulmonary tract and in the gastrointestinal tract, these normal sites of expression are invisible to immune detection as CEA is restricted to the apical surface of the epithelial cells that face the lumen in normal adults [198, 199]. As the cells are ‘invisible’ to immune detection it renders CEA an attractive target with limited bystander cytotoxicity. Following cancer development, epithelial cells lose apical polarity, which subsequently results in CEA gaining access to the blood stream and into the serum of the patient [200]. This renders CEA a useful diagnostic biomarker, as serum detection can serve to identify cancer development for several cancer types including breast [201–203], skin cancer [204], NSCLC [205–207], gastric [202, 208–211], and pancreatic cancer [202, 212–215]. Preclinical testing with CEA-CAR T cells has shown that lymphodepletion or myeloablation prior to infusion is required to induce a response in mice with CEA+ tumors [198]. Initially, CEA was targeted utilizing engineered TCRs, but trials were halted as patients developed severe colitis as a result of off target killing of normal epithelial cells [216]. These same results have yet to be observed with CAR T cell therapy targeting CEA, but patients are treated with caution to avoid on-target, off-tumor cytotoxicity.

### PSMA

Prostate specific membrane antigen (PSMA), or Glutamate carboxypeptidase II (GCPII) [158], is a glycoprotein [217] with three known activities including folate hydrolase [218], NAALADase [219], and dipeptidyl peptidase [217]. While PSMA is expressed in normal prostate epithelium [217], it has been shown in 90% of human prostate tumors including their respective metastatic sites [158, 220, 221]. PSMA has also been expressed in low levels in salivary glands, brain, and kidneys [222–224]. In initial pre-clinical models, anti-PSMA CAR T cells were able to effectively target and eliminate 60% of tumors in treated animals while significantly improving overall survival *in viv o* [225]. Following Phase I clinical trials, no anti-PSMA toxicities were noted and 40% of patients achieved clinical partial responses (PR) [226]. More recently, PSMA CAR T cells have been designed to resist TGFβ suppression, which is commonly found in prostate cancers, via a negative TGFβ receptor II [7]. In patients with castrate metastatic prostate cancer, PSMA-CAR T cell therapy is not only safe, but patients experience cytokine production suggestive of persistence of T cells in the blood for up to 2 weeks (NCT01140373) [227].

### ROR1

Receptor tyrosine kinase like orphan receptor 1 (ROR1) is a Wnt5a surface receptor expressed during embryonic development, but generally absent from adult tissue with the exception of adipocytes, gut, pancreas, and parathyroid glands [228–230]. In the case of cancer, ROR1 has shown high levels in several solid malignancies: pancreatic [231, 232], ovarian [231, 233–235], breast [231, 236–238], lung [231, 239, 240], gastric cancer [241], and colorectal cancer [242]. High levels of ROR1 have shown strong correlation to poor patient outcome and also to developing metastasis [235, 243]. There has been some conflicting preclinical studies where CAR T cells targeting ROR1 have demonstrated severe cytotoxicity as the cells accumulated within the lungs [244]. Meanwhile, other studies have shown great success in targeting ROR1, which may be a direct cause of the specificity of the antibody utilized for the scFv [245, 246]. Currently, ROR1 is being used in clinical trials to target breast and lung cancers.

### FAP

Fibroblast activation protein (FAP) is a transmembrane serine protease with high expression on cancer-associated stromal cells (CASC) in epithelial cancers [247–249]. In pancreatic tumors, FAP shows significant elevation and is correlated with worse clinical outcome [250]. In colorectal cancer, patients with high levels of FAP were more likely to develop metastasis, recurrence, and aggressive disease progression [251]. FAP does not have this same expression within normal cells, as most stromal cells have insignificant levels of the protein [252–254]. As a therapeutic target, FAP has been utilized as a useful cancer vaccine in inhibiting tumor growth and increasing cytotoxicity [247, 255, 256]. As the biomarker has shown success as a targeting agent, CAR T cells targeting FAP have been developed. These FAP CAR T cells show conflicting results as some groups report limited antitumor efficacy [257], while others report significant tumor cytotoxicity with minimal off-tumor killing [258] along with prolonged survival [259]. While the use of FAP CAR T cells may extend to many different organ sites, current clinical trials are designed to treat pleural mesothelioma.

### EpCAM

Epithelial cell adhesion molecule (EpCAM or CD326) is a transmembrane glycoprotein that functions to abrogate E-cadherin-mediated cell adhesion, and functions within transcriptional complexes inducing c-myc and cyclin A & E expression [260, 261]. EpCAM has shown overexpression in a range of tumors including colon adenocarcinoma, stomach adenocarcinoma, pancreatic adenocarcinoma, lung adenocarcinoma, ovarian adenocarcinoma, breast adenocarcinoma, and AML [262–265]. The protein is found at the basolateral cell membrane of normal adult tissue [266]. EpCAM has shown significance as a biomarker for early cancer development [267]. Like several other biomarker targets described, antibody therapy targeting EpCAM (Catumaxomab) has been used in patients to treat peritoneal carcinomatosis (PC) which resulted in a slight increase in survival [268]. Further clinical trials with Catumaxomab have been used to target bladder cancer [269], head and neck cancer [270], ovarian cancer [271], and metastatic disease [272]. These trials resulted in an increase in overall patient survival. EpCAM specific CAR T cells have been developed to treat prostate, breast, and peritoneal cancers and have shown suppressed tumor progression/delayed disease as well as CAR T cell trafficking into the tumor site [273–276].

### EGFRvIII

Epidermal growth factor receptor variant III (EGFRvIII) is a gain of function mutated EGFR that arises from the genomic deletion of exons 2–7. The deletion of these exons leads to a ligand-independent receptor that endows cells with a significant growth advantage over normal cells [277]. EGFRVIII is commonly found within glioblastoma patients, especially in CD133+ glioblastoma cancer stem cells [278]. As a tumor-specific antigen, EGFRvIII has been targeted utilizing FDA approved cancer vaccines (Rindopepimut), which result in significant improved survival [279]. Due to its success as a cancer vaccine, CAR T cells have been developed to directly target malignant cells expressing EGFRvIII. These CAR T cell therapies have shown delayed tumor growth, elimination of EGFRVIII+ tumor cells, and increased pro-inflammatory cytokine release in an antigen dependent manner [280–283]. A first-in-human study of intravenous delivery of a single dose of autologous EGFRvIII-CAR T cells (NCT02209376) had reported that the infusion of cells was feasible and safe, with no off-tumor toxicity or cytokine release syndrome. In this study, 10 patients with recurrent glioblastoma (GBM) were treated with EGFRvIII-CAR T cells. At least one patient achieved stable disease for over 18 months with a single infusion of CAR T cells. The median overall survival was about 8 months in all patients. The study, however, found that tumor microenvironment increased the expression of inhibitory molecules and infiltration by regulatory T cells which suppressed effector CAR T cell functions [284]. While there are promising results using this target, there may be suppressive factors that limit its efficacy in patients. There are nine clinical trials ongoing (as of May 2018) targeting a variety of tumor types.

### EphA2

Ephrin type A receptor (EphA2) is a receptor tyrosine kinase that plays a key role in the development of cancer disease. EphA2 enhances tumorigenesis and progression via interactions with other cell-surface receptors such as EGFR and HER2/ErbB2, which in turn amplify MAPK, Akt, and Rho family GTPase activities [285–287]. EphA2 has shown expression in normal brain, skin, bone marrow, lung, thymus, spleen, liver, small intestine, colon, bladder, kidney, uterus, testis and prostate at low levels [288, 289]. Overexpression of EphA2 has been observed in malignant tissue which has been linked to poor clinical prognosis [290–292]. EphA2 has been targeted through a variety of avenues including viral vectors, RNA interference, small molecule inhibitors, recombinant proteins, and immunotherapy. Small molecule inhibitors (FDA approved-Dasatinib) of EphA2 have significantly reduced tumor growth in several cancer types, and have shown anti-tumor efficacy via the reduction of EphA2 expression and kinase activity upon treatment [293, 294]. On the heels of the success of these methods, CAR T cells have been developed to target EphA2 in Lung cancer [295], glioma [296], and glioblastoma [297] which have all demonstrated cytotoxic effects both in vitro and in vivo [298].

## Combination therapy with multiple biomarker targets

To aid in providing both specificity and longevitiy of CAR T cells, efforts have been made to combine different biomarker targets to elicit T cell responses. Initially designed as enhancers of co-stimulation [299], these CARs are termed “tandem CARs” and are designed to express two antigen binding domains. Following binding of both scFv fragments, CAR T cells are able to send an activation signal and elicit target cell death, but are unable to do this if only one scFv binds [300]. BCMA CAR T cells have been linked to CS1-CAR T cells and designed to express both CAR molecules on the cell surface. They found that this combination elicited potent and specific anti-tumor activity through both antigens in vitro and in vivo [301]. HER2/IL-13RA2 CAR T cells have been designed and showed additive T cell activation when both receptors were engaged, resulting in superior sustained activity [302]. ErbB2/MUC1 CAR T cells have been shown to kill ErbB2 expressing cells efficiently and proliferate in a MUC1 dependent manner [303]. Meanwhile, pan-ErbB CARs are designed to target 8 distinct homo- and hetero-dimers formed by the ErbB network [115]. These tandem CARs avoided antigen escape, which is the primary drawback from CAR therapy as cancer evolves to sequester target antigen expression. CD20/CD19 tandem CARs have also been developed, but showed no difference between tandem CAR killing and single antigen specificity CARs in this context [304]. This demonstrates that only certain combinations of biomarker targets are effective in a tandem CAR design. CD19 has also been combined with Her2 and showed the engineered cells could preserve the cytolytic activity of T cells [305]. This is an ongoing worthwhile pursuit to develop CARs that have specific killing with minimal cytotoxic effects to healthy tissue. By activating upon two ScFv signals, bystander organ killing could be reduced as different antigen combinations can decrease on-target, off-tumor killing. In addition, as another mechanism to enhance CAR efficacy in vivo, CAR T cells are also being constructed to induce transcriptional activation of synthetic notch receptors upon antigen binding. By combining this form of activation with a standard CAR target, cytokine secretion profiles, T cell differentiation, and local delivery of therapeutics can be controlled [306].

In an effort to increase CAR–tumor specificity and reduce off-tumor toxicity inhibitory chimeric antigen receptors (iCARs) have been developed to ensure healthy tissue is not targeted by CAR T cells. iCAR cells are designed with an ingrained override signal. When in contact with only the tumor antigen, CAR T cells elicit a cytotoxic response to the target cell, but when in contact with normal tissue antigens, the T cells are effectively turned ‘off’ via anti-inflammatory co-stimulation. This new technique may provide a way for biomarkers to be used in combination to elicit extremely specific effects within cancer and avoid healthy tissue toxicity [307, 308].

## Up and coming biomarkers

As CAR therapy expands, so does the need for discovering new cancer-specific biomarkers that can serve as targets. We show some biomarkers with preliminary preclinical data that may be useful as future CAR targets.

### CT antigens

Cancer/testis (CT) antigens have normal expression limited to adult testicular germ cells, but have shown expression in various tumor cells such as ovarian cancer, lung cancer, melanoma, breast cancer, glioma, and colon cancer [309–316]. Because male germ cells are unable to present antigens to T cells, CT antigens can be targeted with minimal cytotoxicity to normal tissue. While current efforts to target CT antigens are primarily focused on modified high specific TCR regions [317], there is an opportunity to target these antigens using CAR T cells as well.

### GUCY2C

Guanylyl cyclase C (GUCY2C) is a membrane-bound protein found on the apical surfaces of intestinal epithelial cells, but is also a cancer mucosa antigen that is overexpressed in both primary and metastatic colorectal cancers as well as esophageal and gastric cancers [318–323]. It has been determined that CD8+ T cell responses are expanded when cells are vaccinated against GUCY2C. These cells are effective at eliminating metastatic colorectal tumors [324, 325]. Initial GUCY2C targeting with CAR T cells has shown promising specificity and demonstrated reduced tumor number and increased survival in mice with GUCY2C+ tumors. This target shows potential for the possible CAR T cell treatment of colorectal tumors in human patients.

### TAG-72

Tumor associated glycoprotein-72 (TAG-72) is a pancarcinoma antigen that shows expression in ovarian cancer [326], colorectal cancer [327], breast cancer [328–330], and prostate cancer [331, 332]. While TAG-72 is present in the normal female reproductive tract, the expression is limited and generally weaker than that seen in cancer [333]. While 91% of endometrial adenocarcinoma samples showed TAG-72 expression, the expression of TAG-72 in normal tissue appears to be hormone (estrogen and progesterone) dependent, which can be utilized to prevent expression in normal patient tissue during treatment [334]. As such, TAG-72 may have potential as a possible biomarker for the treatment of some cancer types.

### HPRT1/TK1

Salvage enzymes Thymidine Kinase 1 (TK1) and Hypoxanthine guanine phosphoribosyltransferase (HPRT1) have recently shown potential as surface antigens for CAR T cell therapy. HPRT1 is a salvage pathway enzyme that synthesizes guanine and inosine throughout the cell cycle [335]. The protein is a housekeeping protein that is found within all normal somatic cells in low levels [336]. There is an upregulation of HPRT1 in certain cancer types, making it a promising biomarker for the treatment of these cancers [337, 338]. In addition, the protein has also been shown to have significant surface localization on certain malignancies such as lung and colorectal cancer [339, 340]. As HPRT1 expression is limited to the cytosol within normal cells, the unique surface localization of the protein makes it promising as a targetable biomarker. TK1 is another salvage enzyme responsible for the synthesis of thymidine in the cell cycle and has been used as a serum biomarker for cancer detection and recurrence [341–344]. Recently, there has been evidence that shows that TK1 may also be upregulated within some malignancies and displayed on the surface of the cell [345]. As proteins normally restricted intracellularly, TK1 and HPRT could be used as surface antigens for CAR therapy with minimal bystander cytotoxicity.

## Conclusions

As CAR T cell therapy expands, so does the search for new biomarker targets for both hematological and solid malignancies. We have provided an analysis of the biomarker targets currently under investigation in clinical trials, in addition to those that may show clinical significance in the future upon further development. Immunotherapy is becoming the new standard in patient care and has experienced huge growth and expansion over the last decade. As CAR T cells become more sophisticated and as new biomarkers are discovered to expand treatment to numerous cancer types, the field of immunotherapy will reach more patients and aid in the improvement of care.

## Abbreviations

BCMA: B cell maturation antigen
CD133: Prominin-1
CD19: Cluster of Differentiation 19
CD33: Siglec-3
CD38: Cluster of differentiation 38
CD5: Cluster of differentiation 5
CD70: Cluster of differentiation 70
CEA: Carcinoembryonic antigen
CT antigens: Cancer/testis
EGFRvIII: Epidermal growth factor receptor variant III
EpCam: Epithelial cell adhesion molecule precursor
EphA2: Ephrin type-A receptor 2 precursor
FAP: Fibroblast activation protein alpha
GAP: Ganglioside G2
GPC3: Glypican 3
GUCY2C: Guanylyl cyclase C
Her2: Human Epidermal Growth Factor Receptor 2
HPRT1: Hypoxanthine guanine phosphoribosyltransferase
IL13Rα2: Interleukin 13 receptor, alpha 2
K1: Thymidine Kinase I
MUC1: Mucin 1
PSCA: Prostate stem cell antigen
PSMA: Prostate specific membrane antigen
ROR1: Receptor tyrosine kinase like orphan receptor 1
TAG-72: Tumor associated glycoprotein-72
TME: Tumor microenvironment

## References

[1] Pennock ND, White JT, Cross EW, Cheney EE, Tamburini BA, Kedl RM (2013). T cell responses: naive to memory and everything in between. Adv Physiol Educ.

[2] Cohen IJ, Blasberg R. Impact of the tumor microenvironment on tumor-infiltrating lymphocytes: focus on breast Cancer. Breast Cancer (Auckl). 2017;11:1178223417731565. 10.1177/1178223417731565. 10.1177/1178223417731565 PMC5617083 28979132

[3] Riberdy JM, Mostaghel E, Doyle C (1998). Disruption of the CD4-major histocompatibility complex class II interaction blocks the development of CD4(+) T cells in vivo. Proc Natl Acad Sci U S A.

[4] Finney HM, Akbar AN, Lawson ADG (2004). Activation of resting human primary T cells with chimeric receptors: costimulation from CD28, inducible costimulator, CD134, and CD137 in series with signals from the TCR zeta chain. J Immunol.

[5] Finney HM, Lawson AD, Bebbington CR, Weir AN (1998). Chimeric receptors providing both primary and costimulatory signaling in T cells from a single gene product. J Immunol.

[6] Gross G, Waks T, Eshhar Z (1989). Expression of immunoglobulin-T-cell receptor chimeric molecules as functional receptors with antibody-type specificity. Proc Natl Acad Sci.

[7] Kloss C, Lee J, June C (2016). 638. TGFBeta signaling blockade within PSMA targeted CAR human T cells for the eradication of metastatic prostate Cancer. Mol Ther.

[8] Kochenderfer JN, Dudley ME, Feldman SA, Wilson WH, Spaner DE, Maric I, et al. Cytokine-associated toxicity in a clinical trial of anti-CD19 plenary paper B-cell depletion and remissions of malignancy along with cytokine-associated toxicity in a clinical trial of anti-CD19 chimeric-antigen-receptor – transduced T cells. Blood. 2012;119(12):2709–20. 10.1182/blood-2011-10-384388. 10.1182/blood-2011-10-384388 PMC3327450 22160384

[9] Kochenderfer JN, Dudley ME, Kassim SH, Somerville RPT, Carpenter RO, Maryalice SS (2015). Chemotherapy-refractory diffuse large B-cell lymphoma and indolent B-cell malignancies can be effectively treated with autologous T cells expressing an anti-CD19 chimeric antigen receptor. J Clin Oncol.

[10] Hay KA, Turtle CJ (2017). Chimeric antigen receptor (CAR) T cells: lessons learned from targeting of CD19 in B-cell malignancies. Drugs.

[11] Ruella M, Maus M V. Catch me if you can: leukemia escape after CD19-directed T cell immunotherapies. Comput Struct Biotechnol J. 2016;14:357–362. Natrix Separations. Available from: doi:10.1016/j.csbj.2016.09.003. 10.1016/j.csbj.2016.09.003 PMC5061074 27761200

[12] Jackson H, Brentjens R (2016). Overcoming antigen escape with CART-cell therapy. Cancer Discov.

[13] Lai X, Liu J-Q, Dong L, Ou-Yang H-M, Dian Z-J, Song J-X, et al. CD19 epitope escape after 4SCAR19 T cell therapy resulted in re-establishment of chemo-sensitivity in adult B-cell acute lymphocytic leukemia patients. Blood. 2016;128 Cited 22 May 2018. Available from: http://www.bloodjournal.org/content/128/22/1633?sso-checked=true.

[14] Su W, Yeong KF, Spencer J, Su W, Yeong KF, Spencer J. Immunohistochemical analysis of human CD5 positive B cells : mantle cells and mantle cell lymphoma are not equivalent in terms of CD5 expression short reports Immunohistochemical analysis of human CD5 positive B cells : mantle cells and mantle cell lympho. J Clin Pathol. 2000;53(5):395–7. 10.1136/jcp.53.5.395 PMC1731189 10889824

[15] Doronin II, Vishnyakova PA, Kholodenko IV, Ponomarev ED, Ryazantsev DY, Molotkovskaya IM, et al. T-cell modulatory properties of CD5 and its role in antitumor immune responses. Leukemia. 2007;9:865–77. BioMed Central Ltd. Cited 10 Jan 2018. Available from: https://www.ncbi.nlm.nih.gov/pmc/articles/PMC3583937/.

[16] Filipovich AH, Vallera D, McGlave P, Polich D, Gajl-Peczalska K, Haake R (1990). T cell depletion with anti-CD5 immunotoxin in histocompatible bone marrow transplantation. The correlation between residual CD5 negative T cells and subsequent graft-versus-host disease. Transplantation.

[17] Gasanov SE, Rael ED, Gasanov NE, Vernon LP (1995). In vitro evaluation of Pyrularia thionin-anti-CD5 immunotoxin. Cancer Immunol Immunother.

[18] Antin JH, Bierer BE, Smith BR, Ferrara J, Guinan EC, Sieff C (1991). Selective depletion of bone marrow T lymphocytes with anti-CD5 monoclonal antibodies: effective prophylaxis for graft-versus-host disease in patients with hematologic malignancies. Blood.

[19] Hertler AA, Schlossman DM, Borowitz MJ, Blythman HE, Casellas P, Frankel AE (1989). An anti-CD5 immunotoxin for chronic lymphocytic leukemia: enhancement of cytotoxicity with human serum albumin-monensin. Int J Cancer.

[20] Ravel S, Colombatti M, Casellas P (1992). Internalization and intracellular fate of anti-CD5 monoclonal antibody and anti-CD5 ricin A-chain immunotoxin in human leukemic T cells. Blood.

[21] Manske JM, Buchsbaum DJ, Vallera DA (1989). The role of ricin B chain in the intracellular trafficking of anti-CD5 immunotoxins. J Immunol.

[22] Vallera DA, Manske JM, Buchsbaum J, Azemove SM, Hanna DE (2018). Antigenic modulation by anti-CD5 information about subscribing to the journal of immunology is online at : ANTIGENIC MODULATION BY ANTI-CD5 IMMUNOTOXINS.

[23] Mamonkin M, Rouce RH, Tashiro H, Brenner MK (2015). IMMUNOBIOLOGY a T-cell – directed chimeric antigen receptor for the selective treatment of T-cell malignancies. Blood.

[24] Chen KH, Wada M, Pinz KG, Liu H, Lin KW, Jares A, Firor AE, Shuai X, Salman H, Golightly M, Lan F, Senzel L, Leung EL, Jiang X, Ma Y. Preclinical targeting of aggressive T-cell malignancies using anti-CD5 chimeric antigen receptor. Leukemia. 2017;31(10):2151–60. 10.1038/leu.2017.8 PMC5629371 28074066

[25] MacDonald KP, Munster DJ, Clark GJ, Dzionek A, Schmitz J, Hart DNJ (2002). Characterization of human blood dendritic cell subsets. Cell.

[26] Muñoz L, Nomdedéu JF, López O, Carnicer MJ, Bellido M, Aventín A (2001). Interleukin-3 receptor alpha chain (CD123) is widely expressed in hematologic malignancies. Haematologica.

[27] Shao H, Calvo KR, Grönborg M, Tembhare PR, Kreitman RJ, Stetler-stevenson M, et al. Development and validation of diagnostic criteria. Leuk Res. 2013;37:1–9. Available from: doi:10.1016/j.leukres.2012.11.021. 10.1016/j.leukres.2012.11.021 PMC5575750 23347903

[28] Testa U, Riccioni R, Militi S, Coccia E, Stellacci E, Samoggia P, et al. Associated with enhanced blast proliferation, increased cellularity, and elevated expression of IL-3R ␣ in acute myelogenous leukemia is associated with enhanced blast proliferation, increased cellularity, and poor prognosis. 2011;100:2980–8. 10.1182/blood-2002-03-0852 12351411

[29] Jordan CT, Upchurch D, Szilvassy SJ, Guzman ML, Howard DS, Pettigrew AL (2000). The interleukin-3 receptor alpha chain is a unique marker for human acute myelogenous leukemia stem cells. Leukemia.

[30] Testa U, Pelosi E, Frankel A (2014). CD 123 is a membrane biomarker and a therapeutic target in hematologic malignancies. Biomark Res.

[31] Testa U, Fossati C, Samoggia P, Masciulli R, Mariani G, Hassan HJ (1996). Expression of growth factor receptors in unilineage differentiation culture of purified hematopoietic progenitors. Blood.

[32] Mardiros A, Dos SC (2013). T cells expressing CD123-specific chimeric antigen receptors exhibit specific cytolytic effector functions and antitumor effects against human acute myeloid leukemia. Blood.

[33] Kim E, Ilagan JO, Liang Y, Daubner GM, Lee SC, Ramakrishnan A, Li Y, Chung YR, Micol JB, Murphy ME, Cho H, Kim MK, Zebari AS, Aumann S, Park CY, Buonamici S, Smith PG, Deeg HJ, Lobry C, Aifantis I, Modis Y, Allain FH, Halene S, Bradley RK, Abdel-Wahab O. Mutations Contribute to Myelodysplasia by Mutant-Specific Effects on Exon Recognition. Cancer Cell. 2015;11;27(5):617–30. 10.1016/j.ccell.2015.04.006 PMC4429920 25965569

[34] Fan M, Li M, Gao L, Geng S, Wang J, Wang Y (2017). Chimeric antigen receptors for adoptive T cell therapy in acute myeloid leukemia. J Hematol Oncol.

[35] Tettamanti S, Biondi A, Biagi E, Bonnet D (2014). CD123 AML targeting by chimeric antigen receptors: a novel magic bullet for AML therapeutics?. Oncoimmunology.

[36] Luo Y, Chang L-J, Hu Y, Dong L, Wei G, Huang H. First-in-man CD123-specific chimeric antigen receptor-modified T cells for the treatment of refractory acute myeloid leukemia. Blood. 2015;126 Cited 16 May 2018. Available from: http://www.bloodjournal.org/content/126/23/3778.

[37] Pizzitola I, Anjos-Afonso F, Rouault-Pierre K, Lassailly F, Tettamanti S, Spinelli O (2014). Chimeric antigen receptors against CD33/CD123 antigens efficiently target primary acute myeloid leukemia cells in vivo. Leukemia.

[38] Gill S, Tasian SK, Ruella M, Shestova O, Li Y, Porter DL (2014). Preclinical targeting of human acute myeloid leukemia and myeloablation using chimeric antigen receptor – modi fi ed T cells. Blood.

[39] Walter RB, Gooley TA, Van Der Velden VHJ, Loken MR, Van DJJM, Flowers DA (2007). Brief report CD33 expression and P-glycoprotein – mediated drug efflux inversely correlate and predict clinical outcome in patients with acute myeloid leukemia treated with gemtuzumab ozogamicin monotherapy. Response.

[40] Griffin JD, Linch D, Sabbath K, Larcom P, Schlossman SF (1984). A monoclonal antibody reactive with normal and leukemic human myeloid progenitor cells. Leuk Res.

[41] Dinndorf P, Andrews R, Denis B, Derry R, Wolff L, Bernstein I (2016). Expression of normal myeloid-associated antigens by acute leukemia. Cell.

[42] Schwonzen M, Diehl V, Dellanna M, Staib P (2007). Immunophenotyping of surface antigens in acute myeloid leukemia by flow cytometry after red blood cell lysis. Leuk Res.

[43] Hoyer JD, Grogg KL, Hanson CA, Gamez JD, Dogan A (2008). CD33 detection by immunohistochemistry in paraffin-embedded tissues: a new antibody shows excellent specificity and sensitivity for cells of myelomonocytic lineage. Am J Clin Pathol.

[44] Dutta S, Saxena R (2014). The expression pattern of CD33 antigen can differentiate leukemic from normal progenitor cells in acute myeloid leukemia. Indian J Hematol Blood Transfus.

[45] de Propris MS, Raponi S, Diverio D, Milani ML, Meloni G, Falini B (2011). High CD33 expression levels in acute myeloid leukemia cells carrying the nucleophosmin (NPM1) mutation. Haematologica.

[46] Sievers EL (2001). Efficacy and safety of gemtuzumab ozogamicin in patients with CD33-positive acute myeloid leukaemia in first relapse. Expert Opin Biol Ther.

[47] Rafiq S, Purdon TJ, Schultz LM, Brentjens RJ. CD33-directed chimeric antigen receptor (CAR) T cells for the treatment of acute myeloid leukemia (AML). Blood. 2016;128 Cited 16 May 2018. Available from: http://www.bloodjournal.org/content/128/22/2825?sso-checked=true.

[48] Shaffer DR, Savoldo B, Yi Z, Chow KKH, Kakarla S, Spencer DM (2011). T cells redirected against CD70 for the immunotherapy of CD70-positive malignancies. Blood.

[49] Bowman MR, Crimmins MA, Yetz-Aldape J, Kriz R, Kelleher K, Herrmann S (1994). The cloning of CD70 and its identification as the ligand for CD27. J Immunol.

[50] Hintzen RQ, Lens SM, Beckmann MP, Goodwin RG, Lynch D, van Lier RA (1994). Characterization of the human CD27 ligand, a novel member of the TNF gene family. J Immunol.

[51] McEarchern JA, Smith LM, McDonagh CF, Klussman K, Gordon KA, Morris-Tilden CA (2008). Preclinical characterization of SGN-70, a humanized antibody directed against CD70. Clin Cancer Res.

[52] Baba M, Okamoto M, Hamasaki T, Horai S, Wang X, Ito Y (2008). Highly enhanced expression of CD70 on human T-lymphotropic virus type 1-carrying T-cell lines and adult T-cell leukemia cells. J Virol.

[53] Centre AM (1999). Aberrant expression and reverse signalling of CD70 on malignant B cells.

[54] Hunter ZR, Branagan AR, Santos DD, Tournilhac O, Hatjiharissi E, Xu L, et al. High levels of soluble Immunoregulatory receptors in patients with WaldenströM’s Macroglobulinemia. Blood. 2004;104 Cited 16 May 2018. Available from: http://www.bloodjournal.org/content/104/11/4881?sso-checked=true.

[55] Agathanggelou A, Niedobitek G, Chen R, Nicholls J, Yin W, Youngt LS. Expression of immune regulatory molecules in Epstein-Barr virus-associated nasopharyngeal carcinomas with prominent lymphoid stroma evidence for a functional interaction between epithelial tumor cells and infiltrating lymphoid cells. Am J Pathol. 1995;147 Cited 16 May 2018. Available from: https://www.ncbi.nlm.nih.gov/pmc/articles/PMC1871000/pdf/amjpathol00046-0286.pdf. PMC1871000 7573360

[56] Jin L, Ge H, Long Y, Yang C, Chang YE, Mu L (2018). CD70, a novel target of CAR T-cell therapy for gliomas. Neuro-Oncology.

[57] Miller J, Eisele G, Tabatabai G, Aulwurm S, von Kürthy G, Stitz L (2010). Soluble CD70: a novel immunotherapeutic agent for experimental glioblastoma. J Neurosurg.

[58] Ge H, Mu L, Jin L, Yang C, Chang YE, Long Y (2017). Tumor associated CD70 expression is involved in promoting tumor migration and macrophage infiltration in GBM. Int J Cancer.

[59] Wischhusen J, Jung G, Radovanovic I, Beier C, Steinbach JP, Rimner A (2002). Identification of CD70-mediated apoptosis of immune effector cells as a novel immune escape pathway of human glioblastoma. Cancer Res.

[60] Petrau C, Cornic M, Bertrand P, Maingonnat C, Marchand V, Picquenot JM (2014). CD70: a potential target in breast cancer?. J Cancer.

[61] Jacobs J, Deschoolmeester V, Zwaenepoel K, Rolfo C, Silence K, Rottey S, et al. CD70: an emerging target in cancer immunotherapy. Pharmacol Ther. 2015;155:1–10. Elsevier Inc.. Available from: doi:10.1016/j.pharmthera.2015.07.007. 10.1016/j.pharmthera.2015.07.007 26213107

[62] Adam PJ, Terrett JA, Steers G, Stockwin L, Loader JA, Fletcher GC (2006). CD70 (TNFSF7) is expressed at high prevalence in renal cell carcinomas and is rapidly internalised on antibody binding. Br J Cancer.

[63] Jilaveanu LB, Sznol J, Aziz SA, Duchen D, Kluger HM, Camp RL (2012). CD70 expression patterns in renal cell carcinoma. Hum Pathol.

[64] Junker K, Hindermann W, Voneggeling F, Diegmann J, Haessler K, Schubert J (2005). CD70: a new tumor specific biomarker for renal cell carcinoma. J Urol.

[65] Ryan MC, Kostner H, Gordon KA, Duniho S, Sutherland MK, Yu C (2010). Targeting pancreatic and ovarian carcinomas using the auristatin-based anti-CD70 antibody-drug conjugate SGN-75. Br J Cancer.

[66] American Association for Cancer Research. S, International Cancer Research Foundation. T, William H, Donner Foundation. W, Rosenthal K, Field B, Mesmer D, et al. Cancer research : the official organ of the American Association for Cancer Research, Inc. Membrane proteomic analyses of ovarian cancer identifies the immune modulators CD70 and B7-H2 as candidate markers of cisplatin response. Cancer Res. 2008; Waverly Press. Cited 16 May 2018. Available from: http://cancerres.aacrjournals.org/content/68/9_Supplement/2430.

[67] Aggarwal S, He T, FitzHugh W, Rosenthal K, Feild B, Heidbrink J (2009). Immune modulator CD70 as a potential cisplatin resistance predictive marker in ovarian cancer. Gynecol Oncol.

[68] Wajant H (2016). Therapeutic targeting of CD70 and CD27. Expert Opin Ther Targets.

[69] Nolte MA, van Olffen RW, van Gisbergen KPJM, van Lier RAW (2009). Timing and tuning of CD27-CD70 interactions: the impact of signal strength in setting the balance between adaptive responses and immunopathology. Immunol Rev.

[70] Hendriks J, Gravestein LA, Tesselaar K, van Lier RAW, Schumacher TNM, Borst J (2000). CD27 is required for generation and long-term maintenance of T cell immunity. Nat Immunol.

[71] McEarchern JA, Oflazoglu E, Francisco L, McDonagh CF, Gordon KA, Stone I, Klussman K, Turcott E, van Rooijen N, Carter P, Grewal IS, Wahl AF, Law CL. Engineered anti-CD70 antibody with multiple effector functions exhibits in vitro and in vivo antitumor activities. Blood. 2007;109(3):1185–92. 10.1182/blood-2006-07-034017 17038522

[72] Israel BF, Gulley M, Elmore S, Ferrini S, Feng W, Kenney SC (2005). Anti-CD70 antibodies: a potential treatment for EBV+ CD70-expressing lymphomas. Mol Cancer Ther.

[73] Lokhorst HM, Plesner T, Laubach JP, Nahi H, Gimsing P, Hansson M (2015). Targeting CD38 with Daratumumab monotherapy in multiple myeloma. N Engl J Med.

[74] Deaglio S, Vaisitti T, Billington R, Bergui L, Omede P, Genazzani A (2007). CD38 / CD19 : a lipid raft – dependent signaling complex in human B cells. Blood.

[75] Konopleva M, Estrov Z, Zhao S, Andreeff M, Mehta K (1998). Ligation of cell surface CD38 protein with agonistic monoclonal antibody induces a cell growth signal in myeloid leukemia cells. J Immunol.

[76] Santonocito AM, Consoli U, Bagnato S, Milone G, Palumbo GA, Di Raimondo F (2004). Flow cytometric detection of aneuploid CD38(++) plasmacells and CD19(+) B-lymphocytes in bone marrow, peripheral blood and PBSC harvest in multiple myeloma patients. Leuk Res.

[77] Vences-Catalán F, Santos-Argumedo L (2011). CD38 through the life of a murine B lymphocyte. IUBMB Life.

[78] Dimopoulos MA, Oriol A, Nahi H, San-Miguel J, Bahlis NJ, Usmani SZ (2016). Daratumumab, Lenalidomide, and dexamethasone for multiple myeloma. N Engl J Med.

[79] Mihara K, Yoshida T, Takei Y, Sasaki N, Takihara Y, Kuroda J (2017). T cells bearing anti-CD19 and/or anti-CD38 chimeric antigen receptors effectively abrogate primary double-hit lymphoma cells. J Hematol Oncol.

[80] Carpenter RO, Evbuomwan MO, Pittaluga S, Rose JJ, Raffeld M, Yang S (2013). B-cell maturation antigen is a promising target for adoptive T-cell therapy of multiple myeloma. Clin Cancer Res.

[81] Tai Y-T, Anderson KC (2015). Targeting B-cell maturation antigen in multiple myeloma. Immunotherapy.

[82] Lin L, Xing L, Acharya CM, Wen K, Liu J, Hsieh P, et al. CD8+ anti-BCMA mRNA CAR T-cells effectively kill human multiple myeloma cells in vitro and in vivo. Blood. 2017;130 Cited 22 May 2018. Available from: http://www.bloodjournal.org/content/130/Suppl_1/3067.

[83] Ali SA, Shi V, Maric I, Wang M, Stroncek DF, Rose JJ (2016). T cells expressing an anti-B-cell maturation antigen chimeric antigen receptor cause remissions of multiple myeloma. Blood.

[84] Cohen AD, Garfall AL, Stadtmauer EA, Lacey SF, Lancaster E, Vogl DT, et al. B-cell maturation antigen (BCMA)-specific chimeric antigen receptor T cells (CART-BCMA) for multiple myeloma (MM): initial safety and efficacy from a phase I study. Blood. 2016;128 Cited 22 May 2018. Available from: http://www.bloodjournal.org/content/128/22/1147?sso-checked=true.

[85] Hassan R, Thomas A, Alewine C, Le DT, Jaffee EM, Pastan I (2016). Mesothelin immunotherapy for Cancer: ready for prime time?. J Clin Oncol.

[86] Hassan R, Bera T, Pastan I (2004). Mesothelin: a new target for immunotherapy. Clin Cancer Res.

[87] Hassan R, Remaley AT, Sampson ML, Zhang J, Cox DD, Pingpank J (2006). Detection and quantitation of serum Mesothelin, a tumor marker for patients with mesothelioma and ovarian Cancer. Clin Cancer Res.

[88] Kachala SS, Bograd AJ, Villena-Vargas J, Suzuki K, Servais EL, Kadota K (2014). Mesothelin overexpression is a marker of tumor aggressiveness and is associated with reduced recurrence-free and overall survival in early-stage lung adenocarcinoma. Clin Cancer Res.

[89] Ho M, Bera TK, Willingham MC, Onda M, Hassan R, FitzGerald D (2007). Mesothelin expression in human lung cancer. Clin Cancer Res.

[90] Tchou J, Wang L-C, Selven B, Zhang H, Conejo-Garcia J, Borghaei H (2012). Mesothelin, a novel immunotherapy target for triple negative breast cancer. Breast Cancer Res Treat.

[91] Hassan R, Bera T, Pastan I (2004). Mesothelin: a new target for immunotherapy. Clin Cancer Res.

[92] Rizk NP, Servais EL, Tang LH, Sima CS, Gerdes H, Fleisher M (2012). Tissue and serum mesothelin are potential markers of neoplastic progression in Barrett’s associated esophageal adenocarcinoma. Cancer Epidemiol Biomark Prev.

[93] Einama T, Homma S, Kamachi H, Kawamata F, Takahashi K, Takahashi N (2012). Luminal membrane expression of mesothelin is a prominent poor prognostic factor for gastric cancer. Br J Cancer.

[94] Argani P, Iacobuzio-Donahue C, Ryu B, Rosty C, Goggins M, Wilentz RE (2001). Mesothelin is overexpressed in the vast majority of ductal adenocarcinomas of the pancreas: identification of a new pancreatic cancer marker by serial analysis of gene expression (SAGE). Clin Cancer Res.

[95] Lamberts LE, de Groot DJA, Bense RD, de Vries EGE, Fehrmann RSN (2015). Functional genomic mRNA profiling of a large cancer data base demonstrates mesothelin overexpression in a broad range of tumor types. Oncotarget.

[96] Bera TK, Pastan I (2000). Mesothelin is not required for normal mouse development or reproduction. Mol Cell Biol.

[97] Morello A, Sadelain M, Adusumilli PS (2016). Mesothelin-targeted CARs: driving T cells to solid tumors. Cancer Discov..

[98] Hassan R, Thomas A, Alewine C, Le DT, Jaffee EM, Pastan I (2016). Mesothelin immunotherapy for Cancer: ready for prime time?. J Clin Oncol.

[99] Kachala SS, Bograd AJ, Villena-Vargas J, Suzuki K, Servais EL, Kadota K (2014). Mesothelin overexpression is a marker of tumor aggressiveness and is associated with reduced recurrence-free and overall survival in early-stage lung adenocarcinoma. Clin Cancer Res NIH Public Access.

[100] Nellan A, Rota C, Majzner R, Lester-McCully CM, Griesinger AM, Mulcahy Levy JM (2018). Durable regression of Medulloblastoma after regional and intravenous delivery of anti-HER2 chimeric antigen receptor T cells. J. Immunother. Cancer.

[101] Yarden Y, Sliwkowski MX (2001). Untangling the ErbB signalling network. Nat Rev Mol Cell Biol.

[102] Yan M, Schwaederle M, Arguello D, Millis SZ, Gatalica Z, Kurzrock R (2015). HER2 expression status in diverse cancers: review of results from 37,992 patients. Cancer Metastasis Rev.

[103] Zhu X, Chi F, Wu R, Jin X, Jiang M (2016). HER2 induces cell proliferation and invasion of non-small-cell lung cancer by upregulating COX-2 expression via MEK/ERK signaling pathway. Onco Targets Ther.

[104] Wright C, Nicholson S, Angus B, Sainsbury JR, Farndon J, Cairns J (1992). Relationship between c-erbB-2 protein product expression and response to endocrine therapy in advanced breast cancer. Br J Cancer.

[105] Kumar R, Mandal M, Lipton A, Harvey H, Thompson CB (1996). Overexpression of HER2 modulates bcl-2, bcl-XL, and tamoxifen-induced apoptosis in human MCF-7 breast cancer cells. Clin Cancer Res.

[106] Omabe M, Ahmed S, Sami A, Xie Y, Tao M, Xiang J (2015). HER2-specific vaccines for HER2-positive breast Cancer immunotherapy. World J Vaccines.

[107] Al-Awadhi A, Lee Murray J, Ibrahim NK. Developing anti-HER2 vaccines: breast cancer experience. Int J Cancer. 2018; Wiley-Blackwell. Cited 29 May 2018. Available from: http://doi.wiley.com/10.1002/ijc.31551. 10.1002/ijc.31551 29693245

[108] Perez EA, Romond EH, Suman VJ, Jeong J-H, Sledge G, Geyer CE (2014). Trastuzumab plus adjuvant chemotherapy for human epidermal growth factor receptor 2–positive breast Cancer: planned joint analysis of overall survival from NSABP B-31 and NCCTG N9831. J Clin Oncol.

[109] Han Y, Liu C, Li G, Li J, Lv X, Shi H (2018). Antitumor effects and persistence of a novel HER2 CAR T cells directed to gastric cancer in preclinical models. Am J Cancer Res.

[110] Priceman SJ, Tilakawardane D, Jeang B, Aguilar B, Murad JP, Park AK (2018). Regional delivery of chimeric antigen receptor–engineered T cells effectively targets HER2 ^+^ breast Cancer metastasis to the brain. Clin Cancer Res.

[111] Nellan A, Rota C, Majzner R, Lester-McCully CM, Griesinger AM, Mulcahy Levy JM (2018). Durable regression of Medulloblastoma after regional and intravenous delivery of anti-HER2 chimeric antigen receptor T cells. J Immunother Cancer BioMed Central.

[112] Feng K, Liu Y, Guo Y, Qiu J, Wu Z, Dai H, et al. Phase I study of chimeric antigen receptor modified T cells in treating HER2-positive advanced biliary tract cancers and pancreatic cancers. Protein Cell. 2017; Cited 29 May 2018. Available from: http://www.ncbi.nlm.nih.gov/pubmed/28710747. 10.1007/s13238-017-0440-4 PMC6160389 28710747

[113] Ahmed N, Brawley V, Hegde M, Bielamowicz K, Kalra M, Landi D (2017). HER2-specific chimeric antigen receptor–modified virus-specific T cells for progressive glioblastoma. JAMA Oncol.

[114] Ahmed N, Brawley VS, Hegde M, Robertson C, Ghazi A, Gerken C (2015). Human epidermal growth factor receptor 2 (HER2) –specific chimeric antigen receptor–modified T cells for the immunotherapy of HER2-positive sarcoma. J Clin Oncol.

[115] van Schalkwyk MCI, Papa SE, Jeannon J-P, Urbano TG, Spicer JF, Maher J (2013). Design of a Phase I Clinical Trial to evaluate Intratumoral delivery of ErbB-targeted chimeric antigen receptor T-cells in locally advanced or recurrent head and neck Cancer. Hum Gene Ther Clin Dev.

[116] Papa S, Adami A, Metoudi M, Achkova D, van Schalkwyk M, Parente PA, et al. A phase I trial of T4 CAR T-cell immunotherapy in head and neck squamous cancer (HNSCC). J Clin Oncol. http://ascopubs.org/doi/abs/10.1200/JCO.2018.36.15_suppl.3046.

[117] Cahan LD, Irie RF, Singh R, Cassidenti A, Paulson JC (1982). Identification of a human neuroectodermal tumor antigen (OFA-I-2) as ganglioside GD2. Proc Natl Acad Sci.

[118] Tivnan A, Heilinger T, Ramsey JM, O’Connor G, Pokorny JL, Sarkaria JN (2017). Anti-GD2-ch14.18/CHO coated nanoparticles mediate glioblastoma (GBM)-specific delivery of the aromatase inhibitor, Letrozole, reducing proliferation, migration and chemoresistance in patient-derived GBM tumor cells. Oncotarget.

[119] Alvarez-Rueda N, Desselle A, Cochonneau D, Chaumette T, Clemenceau B, Leprieur S (2011). A monoclonal antibody to O-acetyl-GD2 ganglioside and not to GD2 shows potent anti-tumor activity without peripheral nervous system cross-reactivity. PLoS One.

[120] Yuki N, Yamada M, Tagawa Y, Takahashi H, Handa S (1997). Pathogenesis of the neurotoxicity caused by anti-GD2 antibody therapy. J Neurol Sci.

[121] Schulz G, Cheresh DA, Varki NM, Yu A, Staffileno LK, Reisfeld RA. Detection of ganglioside GD2 in tumor tissues and sera of neuroblastoma patients. Cancer Res. 1984;44(12 Pt 1):5914–20. 6498849

[122] Newick K, Moon E, Albelda SM (2016). Chimeric antigen receptor T-cell therapy for solid tumors. Mol Ther - Oncolytics.

[123] Long AH, Haso WM, Shern JF, Wanhainen KM, Murgai M, Ingaramo M (2015). 4-1BB costimulation ameliorates T cell exhaustion induced by tonic signaling of chimeric antigen receptors. Nat Med.

[124] Pule MA, Savoldo B, Myers GD, Rossig C, Russell HV, Dotti G (2008). Virus-specific T cells engineered to coexpress tumor-specific receptors: persistence and antitumor activity in individuals with neuroblastoma. Nat Med.

[125] Louis CU, Savoldo B, Dotti G, Pule M a, Yvon E, Myers GD, et al. Antitumor activity and long-term fate of chimeric antigen receptor – positive T cells in patients with neuroblastoma. Mol Ther J Am Soc Gene Ther. 2011;14:1324–1334. Available from: doi:10.1038/nm.1882%5Cnhttp://www.ncbi.nlm.nih.gov/pubmed/17299404. 10.1182/blood-2011-05-354449 PMC3234664 21984804

[126] Ploessl C, Pan A, Maples KT, Lowe DK. Dinutuximab: An Anti-GD2 Monoclonal Antibody for High-Risk Neuroblastoma. 2016;50:416–22. Cited 25 May 2018. Available from: http://www.ncbi.nlm.nih.gov/pubmed/26917818. 10.1177/1060028016632013 26917818

[127] Raffaghello L, Marimpietri D, Pagnan G, Pastorino F, Cosimo E, Brignole C (2003). Anti-GD2 monoclonal antibody immunotherapy: a promising strategy in the prevention of neuroblastoma relapse. Cancer Lett.

[128] Ahmed M, Cheung N-KV (2014). Engineering anti-GD2 monoclonal antibodies for cancer immunotherapy. FEBS Lett.

[129] Richman SA, Nunez-Cruz S, Moghimi B, Li LZ, Gershenson ZT, Mourelatos Z (2018). High-affinity GD2-specific CAR T cells induce fatal encephalitis in a preclinical neuroblastoma model. Cancer Immunol Res.

[130] Girling A, Bartkova J, Burchell J, Gendler S, Gillett C, Taylor-Papadimitriou J (1989). A core protein epitope of the polymorphic epithelial mucin detected by the monoclonal antibody SM-3 is selectively exposed in a range of primary carcinomas. Int J Cancer.

[131] van Dam PA, Lowe DG, Watson JV, Jobling TW, Chard T, Shepherd JH (1991). Multi-parameter flow cytometric quantitation of the expression of the tumor-associated antigen SM3 in normal and neoplastic ovarian tissues. A comparison with HMFG1 and HMFG2. Cancer.

[132] Burchell J, Poulsom R, Hanby A, Whitehouse C, Cooper L, Clausen H, et al. An alpha2,3 sialyltransferase (ST3Gal I) is elevated in primary breast carcinomas. Glycobiology. 1999:1307–11. Available from: http://www.ncbi.nlm.nih.gov/pubmed/10561455. 10.1093/glycob/9.12.1307 10561455

[133] Julien S, Picco G, Sewell R, Vercoutter-Edouart AS, Tarp M, Miles D, et al. Sialyl-Tn vaccine induces antibody-mediated tumour protection in a relevant murine model. Br J Cancer. 2009;100:1746–1754. Nature Publishing Group. Available from: doi:10.1038/sj.bjc.6605083. 10.1038/sj.bjc.6605083 PMC2695689 19436292

[134] Brockhausen I, Yang J-M, Burchell J, Whitehouse C, Taylor-Papadimitriou J (1995). Mechanisms underlying aberrant glycosylation of MUC1 mucin in breast Cancer cells. Eur J Biochem.

[135] Lloyd KO, Burchell J, Kudryashov V, Yin BWT, Taylor-Papadimitriou J (1996). Comparison of O -linked carbohydrate chains in MUC-1 mucin from normal breast epithelial cell lines and breast carcinoma cell lines. J Biol Chem.

[136] Hilkens J, Buijs F, Hilgers J, Hageman P, Calafat J, Sonnenberg A (1984). Monoclonal antibodies against human milk-fat globule membranes detecting differentiation antigens of the mammary gland and its tumors. Int J Cancer.

[137] Hayes DF, Sekine H, Ohno T, Abe M, Keefe K, Kufe DW (1985). Use of a murine monoclonal antibody for detection of circulating plasma DF3 antigen levels in breast cancer patients. J Clin Invest.

[138] van de Wiel-van Kemenade E, Ligtenberg MJ, de Boer AJ, Buijs F, Vos HL, Melief CJ (1993). Episialin (MUC1) inhibits cytotoxic lymphocyte-target cell interaction. J Immunol.

[139] Wilkie S, Picco G, Foster J, Davies DM, Julien S, Cooper L (2008). Retargeting of human T cells to tumor-associated MUC1: the evolution of a chimeric antigen receptor. J Immunol.

[140] Maher J, Wilkie S, Davies DM, Arif S, Picco G, Julien S, et al. Targeting of tumor-associated Glycoforms of MUC1 with CAR T cells. Immunity. 2016;45:945–946. Elsevier Inc.. Available from: doi:10.1016/j.immuni.2016.10.014. 10.1016/j.immuni.2016.10.014 27851917

[141] You F, Jiang L, Zhang B, Lu Q, Zhou Q, Liao X (2016). Phase 1 clinical trial demonstrated that MUC1 positive metastatic seminal vesicle cancer can be effectively eradicated by modified anti-MUC1 chimeric antigen receptor transduced T cells. Sci China Life Sci.

[142] Gao H, Li K, Tu H, Pan X, Jiang H, Shi B (2014). Development of T cells redirected to glypican-3 for the treatment of hepatocellular carcinoma. Clin Cancer Res.

[143] Filmus J, Selleck SB (2001). Glypicans: proteoglycans with a surprise. J Clin Invest.

[144] Dargel C, Bassani-Sternberg M, Hasreiter J, Zani F, Bockmann J-H, Thiele F (2015). T cells engineered to express a T-cell receptor specific for Glypican-3 to recognize and kill hepatoma cells in vitro and in mice. Gastroenterology.

[145] Xue R, Feng J, Meng Q, Lv F, Zhu Y, Yu H (2017). The significance of glypican-3 expression profiling in the tumor cellular origin theoretical system for hepatocellular carcinoma progression. J Gastroenterol Hepatol.

[146] Castillo L, Huvelle MAL, Fujita A, Lobba ARM, Tascón R, Garcia TR (2015). Expression of Glypican-3 (GPC3) in malignant and non-malignant human breast tissues. Open Cancer J.

[147] Kandil D, Leiman G, Allegretta M, Evans M (2009). Glypican-3 protein expression in primary and metastatic melanoma: a combined immunohistochemistry and immunocytochemistry study. Cancer Cytopathol.

[148] Mounajjed T, Zhang L, Wu TT. Glypican-3 expression in gastrointestinal and pancreatic epithelial neoplasms. Hum Pathol. 2013;44:542–550. Elsevier Inc.. Available from: doi:10.1016/j.humpath.2012.06.016. 10.1016/j.humpath.2012.06.016 23079207

[149] Baumhoer D, Tornillo L, Stadlmann S, Roncalli M, Diamantis EK, Terracciano LM (2008). Glypican 3 expression in human nonneoplastic, preneoplastic, and neoplastic tissues: a tissue microarray analysis of 4,387 tissue samples. Am J Clin Pathol.

[150] Jiang Z, Jiang X, Chen S, Lai Y, Wei X, Li B (2017). Anti-GPC3-CAR T cells suppress the growth of tumor cells in patient-derived xenografts of hepatocellular carcinoma. Front Immunol.

[151] Zhai B, Shi D, Gao H, Qi X, Jiang H, Zhang Y (2017). A phase I study of anti-GPC3 chimeric antigen receptor modified T cells (GPC3 CAR-T) in Chinese patients with refractory or relapsed GPC3+ hepatocellular carcinoma(r/r GPC3+ HCC). J Clin Oncol.

[152] Papageorgis P, Ozturk S, Lambert AW, Neophytou CM, Tzatsos A, Wong CK, et al. Targeting IL13Ralpha2 activates STAT6-TP63 pathway to suppress breast cancer lung metastasis. Breast Cancer Res. 2015;17:1–15. Available from: doi:10.1186/s13058-015-0607-y. 10.1186/s13058-015-0607-y PMC4531803 26208975

[153] Krebs S, Chow KKH, Yi Z, Rodriguez-Cruz T, Hegde M, Gerken C (2014). T cells redirected to interleukin-13Rα2 with interleukin-13 mutein--chimeric antigen receptors have anti-glioma activity but also recognize interleukin-13Rα1. Cytotherapy.

[154] Brown CE, Badie B, Barish ME, Weng L, Ostberg JR, Chang W-C (2015). Bioactivity and safety of IL13R 2-redirected chimeric antigen receptor CD8+ T cells in patients with recurrent glioblastoma. Clin Cancer Res.

[155] Krenciute G, Krebs S, Torres D, Wu MF, Liu H, Dotti G (2016). Characterization and functional analysis of scFv-based chimeric antigen receptors to redirect T cells to IL13Rα2-positive glioma. Mol Ther.

[156] Brown CE, Alizadeh D, Starr R, Weng L, Wagner JR, Naranjo A (2016). Regression of glioblastoma after chimeric antigen receptor T-cell therapy. N Engl J Med.

[157] Wang MC, Papsidero LD, Kuriyama M, Valenzuela LA, Murphy GP, Chu TM (1981). Prostate antigen: a new potential marker for prostatic cancer. Prostate.

[158] Cunha AC, Weigle B, Kiessling A, Bachmann M, Rieber EP (2006). Tissue-specificity of prostate specific antigens: comparative analysis of transcript levels in prostate and non-prostatic tissues. Cancer Lett.

[159] Ono H, Yanagihara K, Sakamoto H, Yoshida T, Saeki N. Prostate stem cell antigen gene is expressed in islets of pancreas. Anat Cell Biol. 2012;45:149–54. Available from: http://www.pubmedcentral.nih.gov/articlerender.fcgi?artid=3472140&tool=pmcentrez&rendertype=abstract%5Cn. 10.5115/acb.2012.45.3.149 PMC3472140 23094202

[160] Zhigang Z, Wenlv S (2004). Prostate stem cell antigen (PSCA) expression in human prostate cancer tissues and its potential role in prostate carcinogenesis and progression of prostate cancer. World J Surg Oncol.

[161] Gerdts E, Priceman S, Tilakawardane D, Park A, Chang W-C, Wright S (2015). Development and optimization of PSCA-specific CAR T cells for the treatment of bone metastatic prostate cancer. J. Immunother. Cancer.

[162] Gu Z, Thomas G, Yamashiro J, Shintaku IP, Dorey F, Raitano A, Witte ON, Said JW, Loda M, Reiter RE. Prostate stem cell antigen (PSCA) expression increases with high gleason score, advanced stage and bone metastasis in prostate cancer. Oncogene. 2000;19(10):1288–96. 10.1038/sj.onc.1203426 10713670

[163] Zhigang Z, Wenlv S (2004). Prostate stem cell antigen (PSCA) expression in human prostate Cancer tissues: implications for prostate carcinogenesis and progression of prostate Cancer. Jpn J Clin Oncol.

[164] Kiesgen S, Chicaybam L, Chintala NK, Adusumilli PS (2018). Chimeric antigen receptor (CAR) T-cell therapy for thoracic malignancies. J Thorac Oncol.

[165] Zou Q, Yang L, Yang Z, Huang J, Fu X. PSCA and Oct-4 expression in the benign and malignant lesions of gallbladder: implication for carcinogenesis, progression, and prognosis of gallbladder adenocarcinoma. Biomed Res Int. 2013;2013:648420. 10.1155/2013/648420. 10.1155/2013/648420 PMC3747335 23984394

[166] Zhao X, Wang F, Hou M (2016). Expression of stem cell markers nanog and PSCA in gastric cancer and its significance. Oncol Lett.

[167] Wei X, Lai Y, Li J, Qin L, Xu Y, Zhao R, et al. PSCA and MUC1 in non-small-cell lung cancer as targets of chimeric antigen receptor T cells. Oncoimmunology. 2017;6:1–10. Taylor & Francis. Available from: doi:10.1080/2162402X.2017.1284722. 10.1080/2162402X.2017.1284722 PMC5384358 28405515

[168] Abate-Daga D, Lagisetty KH, Tran E, Zheng Z, Gattinoni L, Yu Z (2014). A novel chimeric antigen receptor against prostate stem cell antigen mediates tumor destruction in a humanized mouse model of pancreatic Cancer. Hum Gene Ther.

[169] Anurathapan U, Chan RC, Hindi HF, Mucharla R, Bajgain P, Hayes BC (2014). Kinetics of tumor destruction by chimeric antigen receptor-modified T cells. Mol Ther.

[170] Hillerdal V, Ramachandran M, Leja J, Essand M (2014). Systemic treatment with CAR-engineered T cells against PSCA delays subcutaneous tumor growth and prolongs survival of mice. BMC Cancer.

[171] Wey JS, Stoeltzing O, Ellis LM (2004). Vascular endothelial growth factor receptors: expression and function in solid tumors. Clin Adv Hematol Oncol.

[172] Ellis LM, Hicklin DJ (2008). VEGF-targeted therapy: mechanisms of anti-tumour activity. Nat Rev Cancer.

[173] Dagmara K-M, Mirosław J, Milczek T, Lipińska B, Emerich J. Clinical significance of VEGFR-2 and VEGFR-3 expression in ovarian cancer patients. Polish J Pathol Termedia. Cited 17 May 2018. Available from: https://www.termedia.pl/Journal/-55/Artykul-16683. 21574104

[174] Holzer TR, Fulford AD, Nedderman DM, Umberger TS, Hozak RR, Joshi A (2013). Tumor cell expression of vascular endothelial growth factor receptor 2 is an adverse prognostic factor in patients with squamous cell carcinoma of the lung. PLoS One.

[175] Prewett M, Huber J, Li Y, Santiago A, Connor WO, King K (1999). Antivascular endothelial growth factor receptor ( fetal liver kinase 1 ) monoclonal antibody inhibits tumor angiogenesis and growth of several mouse and human tumors.

[176] Neuchrist C, Erovic BM, Handisurya A, Steiner GE, Rockwell P, Gedlicka C (2001). Vascular endothelial growth factor receptor 2 (VEGFR2) expression in squamous cell carcinomas of the head and neck. Laryngoscope.

[177] O’Byrne KJ, Koukourakis MI, Giatromanolaki A, Cox G, Turley H, Steward WP (2000). Vascular endothelial growth factor, platelet-derived endothelial cell growth factor and angiogenesis in non-small-cell lung cancer. Br J Cancer.

[178] Duff SE, Jeziorska M, Rosa DD, Kumar S, Haboubi N, Sherlock D (2006). Vascular endothelial growth factors and receptors in colorectal cancer: implications for anti-angiogenic therapy. Eur J Cancer.

[179] Price DJ, Miralem T, Jiang S, Steinberg R, Avraham H (2001). Role of vascular endothelial growth factor in the stimulation of cellular invasion and signaling of breast Cancer cells. Cell Growth Differ.

[180] Rydén L, Stendahl M, Jonsson H, Emdin S, Bengtsson NO, Landberg G (2005). Tumor-specific VEGF-A and VEGFR2 in postmenopausal breast cancer patients with long-term follow-up. Implication of a link between VEGF pathway and tamoxifen response. Breast Cancer Res Treat.

[181] Koukourakis MI, Giatromanolaki A, Thorpe PE, Brekken RA, Sivridis E, Kakolyris S (2000). Vascular Endothelial Growth Factor/KDR Activated Microvessel Density versus CD31 Standard Microvessel Density in Non-Small Cell Lung Cancer. Cancer Res.

[182] Hung CJ, Ginzinger DG, Zarnegar R, Kanauchi H, Wong MG, Kebebew E (2003). Expression of vascular endothelial growth factor-C in benign and malignant thyroid tumors. J Clin Endocrinol Metab.

[183] Andersen S, Donnem T, Al-Shibli K, Al-Saad S, Stenvold H, Busund LT (2011). Prognostic impacts of angiopoietins in NSCLC tumor cells and stroma: VEGF-A impact is strongly associated with Ang-2. PLoS One.

[184] Miettinen M, Rikala M-S, Rys J, Lasota J, Wang Z-F (2012). Vascular endothelial growth factor receptor 2 as a marker for malignant vascular tumors and mesothelioma: an immunohistochemical study of 262 vascular endothelial and 1640 nonvascular tumors. Am J Surg Pathol.

[185] Bruns CJ, Liu W, Davis DW, Shaheen RM, McConkey DJ, Wilson MR (2000). Vascular endothelial growth factor is an in vivo survival factor for tumor endothelium in a murine model of colorectal carcinoma liver metastases. Cancer.

[186] Inoue K, Slaton JW, Davis DW, Hicklin DJ, McConkey DJ, Karashima T (2000). Treatment of human metastatic transitional cell carcinoma of the bladder in a murine model with the anti-vascular endothelial growth factor receptor monoclonal antibody DC101 and paclitaxel. Clin Cancer Res.

[187] Camidge DR, Ballas MS, Dubey S, Haigentz M, Rosen PJ, Spicer JF (2010). A phase II, open-label study of ramucirumab (IMC-1121B), an IgG1 fully human monoclonal antibody (MAb) targeting VEGFR-2, in combination with paclitaxel and carboplatin as first-line therapy in patients (pts) with stage IIIb/IV non-small cell lung cancer (NSCLC). J Clin Oncol.

[188] Carvajal RD, Wong MK, Thompson JA, Gordon MS, Lewis KD, Pavlick AC, Wolchok JD, Rojas PB, Schwartz JD, Bedikian AY. A phase 2 randomised study of ramucirumab (IMC-1121B) with or without dacarbazine in patients with metastatic melanoma. Eur J Cancer. 2014;50(12):2099–107. 10.1016/j.ejca.2014.03.289 PMC5702465 24930625

[189] Zhu AX, Finn RS, Mulcahy M, Gurtler J, Sun W, Schwartz JD (2013). A phase II and biomarker study of ramucirumab, a human monoclonal antibody targeting the VEGF receptor-2, as first-line monotherapy in patients with advanced hepatocellular Cancer. Clin Cancer Res.

[190] Clarke JM, Hurwitz HI (2014). Targeted inhibition of VEGF Receptor-2: an update on Ramucirumab. Expert Opin Biol Ther.

[191] Penson RT, Moore KM, Fleming GF, Braly P, Schimp V, Nguyen H, Matulonis UA, Banerjee S, Haluska P, Gore M, Bodurka DC, Hozak RR, Joshi A, Xu Y, Schwartz JD, McGuire WP. A phase II study of ramucirumab (IMC-1121B) in the treatment of persistent or recurrent epithelial ovarian, fallopian tube or primary peritoneal carcinoma. Gynecol Oncol. 2014;134(3):478–85. 10.1016/j.ygyno.2014.06.029 PMC5166425 25016924

[192] Cancer Immunotherapy- Clinical Trials.gov. Available from: https://clinicaltrials.gov/ct2/results?cond=Cancer&term=cancer+immunotherapy&cntry=&state=&city=&dist=.

[193] Li K, Lan Y, Wang J, Liu L. Chimeric antigen receptor – engineered T cells for liver cancers, progress and obstacles. Tumor Biol. 2017:1–8. 10.1177/1010428317692229 28347250

[194] Burga RA, Thorn M, Point GR, Guha P, Nguyen CT, Licata LA (2015). Liver myeloid-derived suppressor cells expand in response to liver metastases in mice and inhibit the anti-tumor efficacy of anti-CEA CAR-T. Cancer Immunol Immunother.

[195] Moreno Carretero G, Cerdán Miguel FJ, Maestro de las Casas ML, Martínez Cortijo S, Ortega MD, Pardo Martínez M (1998). Serum and tissue CEA in colorectal cancer: clinical relevance. Rev Esp Enferm Dig.

[196] Ng IO, Ho J, Pritchett CJ, Chan EY, Ho FC (1993). CEA tissue staining in colorectal cancer patients--correlation with plasma CEA, histology and staging. Pathology.

[197] Fichera A, Michelassi F, Arenas RB (1998). Selective expression of carcinoembryonic antigen promoter in cancer cell lines: targeting strategy for gene therapy in colorectal cancer. Dis Colon Rectum.

[198] Wang L, Ma N, Okamoto S, Amaishi Y, Sato E, Seo N, et al. Efficient tumor regression by adoptively transferred CEA-specific CAR-T cells associated with symptoms of mild cytokine release syndrome. Oncoimmunology. 2016;5:1–13. Taylor & Francis. Available from: doi:10.1080/2162402X.2016.1211218. 10.1080/2162402X.2016.1211218 PMC5048773 27757303

[199] Nap M, Mollgard K, Burtin P, Fleuren GJ (1988). Immunohistochemistry of carcino-embryonic antigen in the embryo, fetus and adult. Tumour Biol.

[200] Yan Z, Deng X, Chen M, Xu Y, Ahram M, Sloane BF (1997). Oncogenic c-Ki-ras but not oncogenic c-ha-ras up-regulates CEA expression and disrupts basolateral polarity in colon epithelial cells. J Biol Chem.

[201] Shao Y, Sun X, He Y, Liu C, Liu H (2015). Elevated levels of serum tumor markers CEA and CA15-3 are prognostic parameters for different molecular subtypes of breast cancer. PLoS One.

[202] Nan J, Li J, Li X, Guo G, Wen X, Tian Y. Preoperative serum carcinoembryonic antigen as a marker for predicting the outcome of three cancers. Biomark Cancer. 2017;9:1–7. Available from: http://www.pubmedcentral.nih.gov/articlerender.fcgi?artid=PMC5345947. 10.1177/1179299X17690142 PMC5345947 28469484

[203] Kazarian A, Blyuss O, Metodieva G, Gentry-Maharaj A, Ryan A, Kiseleva EM, et al. Testing breast cancer serum biomarkers for early detection and prognosis in pre-diagnosis samples. Br J Cancer. 2017;116:501–508. Nature Publishing Group. Available from: doi:10.1038/bjc.2016.433. 10.1038/bjc.2016.433 PMC5318971 28081538

[204] Latteri S, Catania VE, Malaguarnera G, Peri A, Bertino G, Frazzetto G (2018). Carcinoembryonic antigen serum levels in nonmelanoma skin cancer. Biomedicine.

[205] Dong Y, Zheng X, Yang Z, Sun M, Zhang G, An X (2016). Serum carcinoembryonic antigen, neuron-specific enolase as biomarkers for diagnosis of nonsmall cell lung cancer. J Cancer Res Ther.

[206] Arrieta O, Morales M, Dorantes-Gallareta Y, Pena O, Martínez-Barrera L, Morales-Espinosa D, et al. Usefulness of serum carcinoembryonic antigen (CEA) monitoring to define response or progression to chemotherapy and its correlation with survival in patients with advanced non small-cell lung cancer: a prospective cohort study. J Thorac Oncol. 2011;6:S1105. Available from: https://bmccancer.biomedcentral.com/articles/10.1186/1471-2407-13-254.

[207] Kataoka Y, Hirano K, Narabayashi T, Hara S, Fujimoto D, Tanaka T (2018). Carcinoembryonic antigen as a predictive biomarker of response to Nivolumab in non–small cell lung Cancer. Anticancer Res.

[208] Jin Z, Jiang W, Wang L (2015). Biomarkers for gastric cancer: progression in early diagnosis and prognosis (review). Oncol Lett.

[209] Căinap C, Nagy V, Gherman A, Cetean S, Laszlo I, Constantin A-M (2015). Classic tumor markers in gastric Cancer. Current standards and limitations. Clujul Med.

[210] Shimada H, Noie T, Ohashi M, Oba K, Takahashi Y (2014). Clinical significance of serum tumor markers for gastric cancer: a systematic review of literature by the task force of the Japanese gastric Cancer association. Gastric Cancer.

[211] Ahn HS, Shin YS, Park PJ, Kang KN, Kim Y, Lee HJ, et al. Serum biomarker panels for the diagnosis of gastric adenocarcinoma. Br J Cancer. 2012;106:733–739. Nature Publishing Group. Available from: doi:10.1038/bjc.2011.592. 10.1038/bjc.2011.592 PMC3322950 22240791

[212] Meng Q, Shi S, Liang C, Liang D, Xu W, Ji S (2017). Diagnostic and prognostic value of carcinoembryonic antigen in pancreatic cancer: a systematic review and meta-analysis. Onco. Targets. Ther.

[213] Chang JC, Kundranda M (2017). Novel diagnostic and predictive biomarkers in pancreatic adenocarcinoma. Int J Mol Sci.

[214] Bhat K, Wang F, Ma Q, Li Q, Mallik S, Hsieh T (2012). Advances in biomarker research for pancreatic Cancer. Curr Pharm Des.

[215] Zeng Z, Cohen AM, Urmacher C (1993). Usefulness of carcinoembryonic antigen monitoring despite normal preoperative values in node-positive colon cancer patients. Dis Colon Rectum.

[216] Parkhurst MR, Yang JC, Langan RC, Dudley ME, Nathan DAN, Feldman SA, et al. T cells targeting carcinoembryonic antigen can mediate regression of metastatic colorectal cancer but induce severe transient colitis. Mol Ther. 2011;19:620–626. The American Society of Gene & Cell Therapy. Available from: doi:10.1038/mt.2010.272. 10.1038/mt.2010.272 PMC3048186 21157437

[217] Schmittgen TD, Teske S, Vessella RL, True LD, Zakrajsek BA (2003). Expression of prostate specific membrane antigen and three alternatively spliced variants of PSMA in prostate cancer patients. Int J Cancer.

[218] Pinto JT, Suffoletto BP, Berzin TM, Qiao CH, Lin S, Tong WP (1996). Prostate-specific membrane antigen: a novel folate hydrolase in human prostatic carcinoma cells. Clin Cancer Res.

[219] Carter RE, Feldman AR, Coyle JT (1996). Prostate-specific membrane antigen is a hydrolase with substrate and pharmacologic characteristics of a neuropeptidase. Proc Natl Acad Sci.

[220] Kiessling A, Wehner R, Füssel S, Bachmann M, Wirth MP, Schmitz M (2012). Tumor-associated antigens for specific immunotherapy of prostate cancer. Cancers (Basel).

[221] Feldmann A, Arndt C, Bergmann R, Loff S, Cartellieri M, Bachmann D (2017). Retargeting of T lymphocytes to PSCA- or PSMA positive prostate cancer cells using the novel modular chimeric antigen receptor platform technology &amp;#x201C;UniCAR&amp;#x201D. Oncotarget.

[222] Kawakami M, Nakayama J (1997). Enhanced expression of the prostate specific membrane antigen gene in prostate cancer as revealed by in situ hybridization. Cancer Res.

[223] Mhawech-Fauceglia P, Zhang S, Terracciano L, Sauter G, Chadhuri A, Herrmann FR (2007). Prostate-specific membrane antigen (PSMA) protein expression in normal and neoplastic tissues and its sensitivity and specificity in prostate adenocarcinoma: an immunohistochemical study using mutiple tumour tissue microarray technique. Histopathology.

[224] Feldmann A, Arndt C, Bergmann R, Loff S, Cartellieri M, Bachmann D (2017). Retargeting of T lymphocytes to PSCA- or PSMA positive prostate cancer cells using the novel modular chimeric antigen receptor platform technology &amp;#x201C;UniCAR&amp;#x201D. Oncotarget.

[225] Zuccolotto G, Fracasso G, Merlo A, Montagner IM, Rondina M, Bobisse S, Figini M, Cingarlini S, Colombatti M, Zanovello P, Rosato A. PSMA-specific CAR-engineered T cells eradicate disseminated prostate cancer in preclinical models. PLoS One. 20143;9(10):e109427. 10.1371/journal.pone.0109427 PMC4184866 25279468

[226] Junghans RP, Ma Q, Rathore R, Gomes EM, Bais AJ, Lo ASY (2016). Phase I trial of anti-PSMA designer CAR-T cells in prostate Cancer: possible role for interacting interleukin 2-T cell pharmacodynamics as a determinant of clinical response. Prostate.

[227] Slovin SF, Wang X, Hullings M, Arauz G, Bartido S, Lewis JS (2013). Chimeric antigen receptor (CAR ^+^ ) modified T cells targeting prostate-specific membrane antigen (PSMA) in patients (pts) with castrate metastatic prostate cancer (CMPC). J Clin Oncol.

[228] Borcherding N, Kusner D, Liu GH, Zhang W (2014). ROR1, an embryonic protein with an emerging role in cancer biology. Protein Cell.

[229] Zhou J-K, Zheng Y-Z, Liu X-S, Gou Q, Ma R, Guo C-L (2017). ROR1 expression as a biomarker for predicting prognosis in patients with colorectal cancer. Oncotarget.

[230] Hojjat-Farsangi M, Moshfegh A, Daneshmanesh AH, Khan AS, Mikaelsson E, Österborg A (2014). The receptor tyrosine kinase ROR1 – an oncofetal antigen for targeted cancer therapy. Semin Cancer Biol.

[231] Balakrishnan A, Goodpaster T, Randolph-Habecker J, Hoffstrom BG, Jalikis FG, Koch LK (2017). Analysis of ROR1 protein expression in human cancer and normal tissues. Clin Cancer Res.

[232] Zhang S, Chen L, Wang-Rodriguez J, Zhang L, Cui B, Frankel W (2012). The onco-embryonic antigen ROR1 is expressed by a variety of human cancers. Am J Pathol.

[233] Henry CE, Emmanuel C, Lambie N, Loo C, Kan B, Kennedy CJ, et al. Distinct patterns of stromal and tumor expression of ROR1 and ROR2 in histological subtypes of epithelial ovarian Cancer. Transl Oncol 2017;10:346–356. The Authors. Available from: doi:10.1016/j.tranon.2017.01.014. 10.1016/j.tranon.2017.01.014 PMC5367847 28342318

[234] Tan H, He Q, Gong G, Wang Y, Li J, Wang J (2016). MiR 382 inhibits migration and invasion by targeting ROR1 through regulating EMT in ovarian cancer. Int J Oncol.

[235] Zhang H, Qiu J, Ye C, Yang D, Gao L, Su Y (2014). ROR1 expression correlated with poor clinical outcome in human ovarian cancer. Sci Rep.

[236] Li C, Wang S, Xing Z, Lin A, Liang K, Song J, Hu Q, Yao J, Chen Z, Park PK, Hawke DH, Zhou J, Zhou Y, Zhang S, Liang H, Hung MC, Gallick GE, Han L, Lin C, Yang L. A ROR1-HER3-lncRNA signalling axis modulates the Hippo-YAP pathway to regulate bone metastasis. Nat Cell Biol. 2017;19(2):106–19. 10.1038/ncb3464 PMC5336186 28114269

[237] Chien H-P, Ueng S-H, Chen S-C, Chang Y-S, Lin Y-C, Lo Y-F (2016). Expression of ROR1 has prognostic significance in triple negative breast cancer. Virchows Arch.

[238] Zhang S, Chen L, Cui B, Chuang H-Y, Yu J, Wang-Rodriguez J (2012). ROR1 is expressed in human breast Cancer and associated with enhanced tumor-cell growth. PLoS One.

[239] Zheng Y-Z, Ma R, Zhou J-K, Guo C-L, Wang Y-S, Li Z-G (2016). ROR1 is a novel prognostic biomarker in patients with lung adenocarcinoma. Sci Rep.

[240] Liu Y, Yang H, Chen T, Luo Y, Xu Z, Li Y (2015). Silencing of receptor tyrosine kinase ROR1 inhibits tumor-cell proliferation via PI3K/AKT/mTOR signaling pathway in lung adenocarcinoma. PLoS One.

[241] Chang H, Jung WY, Kang Y, Lee H, Kim A, Kim B (2015). Expression of ROR1, pAkt, and pCREB in gastric adenocarcinoma. Ann Diagn Pathol.

[242] Zhou J-K, Zheng Y-Z, Liu X-S, Gou Q, Ma R, Guo C-L (2017). ROR1 expression as a biomarker for predicting prognosis in patients with colorectal cancer. Oncotarget.

[243] Henry C, Llamosas E, Knipprath-Meszaros A, Schoetzau A, Obermann E, Fuenfschilling M (2015). Targeting the ROR1 and ROR2 receptors in epithelial ovarian cancer inhibits cell migration and invasion. Oncotarget.

[244] Rottman JB, Ganley K, Daly B, Horton HM, Friedman KM, Perkins M, et al. ROR1-directed chimeric antigen receptor T cell recognition of self-antigen is associated with acute toxicity, T cell dysfunction, and poor tumor control. Blood. 2017;130:4450.

[245] Gohil S, Paredes-Moscosso S, Harrasser M, Davidoff A, Pule M, Della Peruta M (2017). Preclinical development of novel humanised ROR1 targeting chimeric antigen receptor T cells and bispecific T-cell engagers. Lancet.

[246] Gohil SH, Paredes-Moscosso SR, Harrasser M, Vezzalini M, Scarpa A, Morris E (2017). An ROR1 bi-specific T-cell engager provides effective targeting and cytotoxicity against a range of solid tumors. Oncoimmunology.

[247] Wang LC, Lo A, Scholler J, Sun J, Majumdar RS, Kapoor V, Antzis M, Cotner CE, Johnson LA, Durham AC, Solomides CC, June CH, Puré E, Albelda SM. Targeting fibroblast activation protein in tumor stroma with chimeric antigen receptor T cells can inhibit tumor growth and augment host immunity without severe toxicity. Cancer Immunol Res. 2014;2(2):154–66. 10.1158/2326-6066.CIR-13-0027 PMC4007316 24778279

[248] Goscinski MA, Suo Z, Flørenes VA, Vlatkovic L, Nesland JM, Giercksky K-E (2008). FAP-α and uPA show different expression patterns in premalignant and malignant esophageal lesions. Ultrastruct Pathol.

[249] Scanlan MJ, Raj BK, Calvo B, Garin-Chesa P, Sanz-Moncasi MP, Healey JH (1994). Molecular cloning of fibroblast activation protein alpha, a member of the serine protease family selectively expressed in stromal fibroblasts of epithelial cancers. Proc Natl Acad Sci.

[250] Cohen SJ, Alpaugh RK, Palazzo I, Meropol NJ, Rogatko A, Xu Z (2008). Fibroblast activation protein and its relationship to clinical outcome in pancreatic adenocarcinoma. Pancreas.

[251] Henry LR, Lee HO, Lee JS, Klein-Szanto A, Watts P, Ross EA (2007). Clinical implications of fibroblast activation protein in patients with colon cancer. Clin Cancer Res.

[252] Rerrig WJ, Garin-Chesat P, Beresford HR, Oettgen HF, Melamedt MR, Old LJ (1988). Cell-surface glycoproteins of human sarcomas: differential expression in normal and malignant tissues and cultured cells. Proc Natl Acad Sci.

[253] Huber MA, Schubert RD, Peter RU, Kraut N, Park JE, Rettig WJ (2003). Fibroblast activation protein: differential expression and serine protease activity in reactive stromal fibroblasts of melanocytic skin tumors. J Invest Dermatol.

[254] Aertgeerts K, Levin I, Shi L, Snell GP, Jennings A, Prasad GS (2005). Structural and kinetic analysis of the substrate specificity of human fibroblast activation protein alpha. J Biol Chem.

[255] Loeffler M, Krüger JA, Niethammer AG, Reisfeld RA (2006). Targeting tumor-associated fibroblasts improves cancer chemotherapy by increasing intratumoral drug uptake. J Clin Invest.

[256] Ostermann E, Garin-Chesa P, Heider KH, Kalat M, Lamche H, Puri C (2008). Effective immunoconjugate therapy in cancer models targeting a serine protease of tumor fibroblasts. Clin Cancer Res.

[257] Tran E, Chinnasamy D, Yu Z, Morgan RA, Lee C-CR, Restifo NP (2013). Immune targeting of fibroblast activation protein triggers recognition of multipotent bone marrow stromal cells and cachexia. J Exp Med.

[258] Lee PP, Yee C, Savage PA, Fong L, Brockstedt D, Weber JS (1999). Characterization of circulating T cells specific for tumor-associated antigens in melanoma patients. Nat Med.

[259] Schuberth PC, Hagedorn C, Jensen SM, Gulati P, van den Broek M, Mischo A (2013). Treatment of malignant pleural mesothelioma by fibroblast activation protein-specific re-directed T cells. J Transl Med.

[260] van der Gun BTF, Melchers LJ, Ruiters MHJ, de Leij LFMH, McLaughlin PMJ, Rots MG (2010). EpCAM in carcinogenesis: the good, the bad or the ugly. Carcinogenesis.

[261] Schnell U, Cirulli V, Giepmans BNG. EpCAM: structure and function in health and disease. Biochim Biophys Acta Biomembr. 2013;1828:1989–2001. Elsevier B.V.. Available from: doi:10.1016/j.bbamem.2013.04.018. 10.1016/j.bbamem.2013.04.018 23618806

[262] Balzar M, Winter MJ, de Boer CJ, Litvinov SV (1999). The biology of the 17-1A antigen (ep-CAM). J Mol Med (Berl).

[263] Zheng X, Fan X, Fu B, Zheng M, Zhang A, Zhong K (2017). EpCAM inhibition sensitizes Chemoresistant leukemia to immune surveillance. Cancer Res.

[264] Xia Ang W, Li Z, Chi Z, Du S-H, Chen C, Tay JCK (2017). Intraperitoneal immunotherapy with T cells stably and transiently expressing anti-EpCAM CAR in xenograft models of peritoneal carcinomatosis. Oncotarget.

[265] Patriarca C, Macchi RM, Marschner AK, Mellstedt H. Epithelial cell adhesion molecule expression (CD326) in cancer: a short review. Cancer Treat Rev. 2012;38:68–75. Elsevier Ltd. Available from: doi:10.1016/j.ctrv.2011.04.002. 10.1016/j.ctrv.2011.04.002 21576002

[266] Patriarca C, Macchi RM, Marschner AK, Mellstedt H (2012). Epithelial cell adhesion molecule expression (CD326) in cancer: a short review. Cancer Treat Rev.

[267] Litvinov SV, van Driel W, van Rhijn CM, Bakker HA, van Krieken H, Fleuren GJ (1996). Expression of ep-CAM in cervical squamous epithelia correlates with an increased proliferation and the disappearance of markers for terminal differentiation. Am J Pathol.

[268] Heiss MM, Murawa P, Koralewski P, Kutarska E, Kolesnik OO, Ivanchenko VV (2010). The trifunctional antibody catumaxomab for the treatment of malignant ascites due to epithelial cancer: results of a prospective randomized phase II/III trial. Int J Cancer.

[269] Kowalski M, Entwistle J, Cizeau J, Niforos D, Loewen S, Chapman W (2010). A phase i study of an intravesically administered immunotoxin targeting EpCAM for the treatment of nonmuscle-invasive bladder cancer in BCG-refractory and BCG-intolerant patients. Drug Des Devel Ther.

[270] GC MD, Rasamoelisolo M, Entwistle J, Cizeau J, Bosc D, Cuthbert W (2009). A phase I clinical study of VB4-845: weekly intratumoral administration of an anti-EpCAM recombinant fusion protein in patients with squamous cell carcinoma of the head and neck. Drug Des. Devel. Ther..

[271] Berek JS, Edwards RP, Parker LP, DeMars LR, Herzog TJ, Lentz SS (2014). Catumaxomab for the treatment of malignant ascites in patients with chemotherapy-refractory ovarian Cancer. Int J Gynecol Cancer.

[272] Andersson Y, Engebraaten O, Juell S, Aamdal S, Brunsvig P, Fodstad (2015). Phase I trial of EpCAM-targeting immunotoxin MOC31PE, alone and in combination with cyclosporin. Br J Cancer.

[273] Tavri S, Jha P, Meier R, Henning TD, Müller T, Hostetter D (2009). Optical imaging of cellular immunotherapy against prostate Cancer. Mol Imaging.

[274] Shirasu N, Yamada H, Shibaguchi H, Kuroki M, Kuroki M (2012). Molecular characterization of a fully human chimeric T-cell antigen receptor for tumor-associated antigen EpCAM. J Biomed Biotechnol.

[275] Deng Z, Wu Y, Ma W, Zhang S, Zhang YQ (2015). Adoptive T-cell therapy of prostate cancer targeting the cancer stem cell antigen EpCAM. BMC Immunol.

[276] Xia Ang W, Li Z, Chi Z, Du S-H, Chen C, Tay JCK (2017). Intraperitoneal immunotherapy with T cells stably and transiently expressing anti-EpCAM CAR in xenograft models of peritoneal carcinomatosis. Oncotarget.

[277] Greenall SA, Johns TG (2016). EGFRvIII: the promiscuous mutation. Cell Death Discov.

[278] Warren JJ, Blanchette D (2017). Dawson D V, Teresa a, Phipps KR, Starr D, et al. Targeting a glioblastoma cancer stem cell population defined by EGF receptor variant III.

[279] Swartz AM, Li Q-J, Sampson JH (2014). Rindopepimut: a promising immunotherapeutic for the treatment of glioblastoma multiforme. Immunotherapy.

[280] Ohno M, Natsume A, Ichiro Iwami K, Iwamizu H, Noritake K, Ito D (2010). Retrovirally engineered T-cell-based immunotherapy targeting type III variant epidermal growth factor receptor, a glioma-associated antigen. Cancer Sci.

[281] Johnson LA, Scholler J, Ohkuri T, Kosaka A, Patel PR, McGettigan SE, Nace AK, Dentchev T, Thekkat P, Loew A, Boesteanu AC, Cogdill AP, Chen T, Fraietta JA, Kloss CC, Posey AD Jr, Engels B, Singh R, Ezell T, Idamakanti N, Ramones MH, Li N, Zhou L, Plesa G, Seykora JT, Okada H, June CH, Brogdon JL, Maus MV. Rational development and characterization of humanized anti-EGFR variant III chimeric antigen receptor T cells for glioblastoma. Sci Transl Med. 2015;7(275):275ra22. 10.1126/scitranslmed.aaa4963 PMC4467166 25696001

[282] Shen CJ, Yang YX, Han EQ, Cao N, Wang YF, Wang Y (2013). Chimeric antigen receptor containing ICOS signaling domain mediates specific and efficient antitumor effect of T cells against EGFRvIII expressing glioma. J Hematol Oncol.

[283] Ohno M, Ohkuri T, Kosaka A, Tanahashi K, June CH, Natsume A (2013). Expression of miR-17-92 enhances anti-tumor activity of T-cells transduced with the anti-EGFRvIII chimeric antigen receptor in mice bearing human GBM xenografts. J Immunother Cancer.

[284] O’Rourke DM, Nasrallah MP, Desai A, Melenhorst JJ, Mansfield K, Morrissette JJD (2017). A single dose of peripherally infused EGFRvIII-directed CAR T cells mediates antigen loss and induces adaptive resistance in patients with recurrent glioblastoma. Sci Transl Med.

[285] Song W, Hwang Y, Youngblood VM, Cook RS, Balko JM, Chen J, et al. Targeting EphA2 impairs cell cycle progression and growth of basal-like/triple-negative breast cancers. Oncogene. 2017;36:5620–5630. Nature Publishing Group. Available from: doi:10.1038/onc.2017.170. 10.1038/onc.2017.170 PMC5629103 28581527

[286] Brantley-Sieders DM, Zhuang G, Hicks D, Wei BF, Hwang Y, Cates JMM (2008). The receptor tyrosine kinase EphA2 promotes mammary adenocarcinoma tumorigenesis and metastatic progression in mice by amplifying ErbB2 signaling. J Clin Invest.

[287] Larsen AB, Pedersen MW, Stockhausen M-T, Grandal MV, van Deurs B, Poulsen HS (2007). Activation of the EGFR gene target EphA2 inhibits epidermal growth factor-induced Cancer cell motility. Mol Cancer Res.

[288] Hafner C, Schmitz G, Meyer S, Bataille F, Hau P, Langmann T (2004). Differential gene expression of Eph receptors and Ephrins in benign human tissues and cancers. Clin Chem.

[289] Park J, Son A, Zhou R (2013). Roles of EphA2 in development and disease. Genes (Basel).

[290] Andres AC, Reid HH, Zürcher G, Blaschke RJ, Albrecht D, Ziemiecki A (1994). Expression of two novel eph-related receptor protein tyrosine kinases in mammary gland development and carcinogenesis. Oncogene.

[291] Andres AC, Zuercher G, Djonov V, Flueck M, Ziemiecki A (1995). Protein tyrosine kinase expression during the estrous cycle and carcinogenesis of the mammary gland. Int J Cancer.

[292] Pasquale EB (2010). Eph receptors and ephrins in cancer: bidirectional signalling and beyond. Nat Rev Cancer.

[293] Huang F, Reeves K, Han X, Fairchild C, Platero S, Wong TW (2007). Identification of candidate molecular markers predicting sensitivity in solid tumors to dasatinib: rationale for patient selection. Cancer Res.

[294] Tandon M1, Vemula SV, Mittal SK. Emerging strategies for EphA2 receptor targeting for cancer therapeutics. Expert Opn Ther Targets. 2011;15(1):31–51. 10.1517/14728222.2011.538682 PMC3016619 21142802

[295] Li N, Liu S, Sun M, Chen W, Xu X, Zeng Z (2018). Chimeric antigen receptor-modified T cells redirected to EphA2 for the immunotherapy of non-small cell lung Cancer. Transl Oncol.

[296] Yi Z, Prinzing BL, Cao F, Gottschalk S, Krenciute G (2018). Optimizing EphA2-CAR T cells for the adoptive immunotherapy of glioma. Mol Ther - Methods Clin Dev.

[297] Chow KK, Naik S, Kakarla S, Brawley VS, Shaffer DR, Yi Z (2013). T cells redirected to EphA2 for the immunotherapy of glioblastoma. Mol Ther.

[298] Song W, Hwang Y, Youngblood VM, Cook RS, Balko JM, Chen J (2017). Targeting EphA2 impairs cell cycle progression and growth of basal-like/triple-negative breast cancers. Oncogene.

[299] Alvarez-Vallina L, Hawkins RE (1996). Antigen-specific targeting of CD28-mediated T cell co-stimulation using chimeric single-chain antibody variable fragment-CD28 receptors. Eur J Immunol.

[300] Wang Y, Luo F, Yang J, Zhao C, Chu Y (2017). New chimeric antigen receptor Design for Solid Tumors. Front Immunol.

[301] Chen KH, Wada M, Pinz KG, Liu H, Shuai X, Chen X (2018). A compound chimeric antigen receptor strategy for targeting multiple myeloma. Leukemia.

[302] Hegde M, Mukherjee M, Grada Z, Pignata A, Landi D, Navai SA (2016). Tandem CAR T cells targeting HER2 and IL13Rα2 mitigate tumor antigen escape. J Clin Invest.

[303] Wilkie S, van Schalkwyk MCI, Hobbs S, Davies DM, van der Stegen SJC, Pereira ACP (2012). Dual targeting of ErbB2 and MUC1 in breast Cancer using chimeric antigen receptors engineered to provide complementary signaling. J Clin Immunol.

[304] Schneider D, Xiong Y, Wu D, Nölle V, Schmitz S, Haso W (2017). A tandem CD19/CD20 CAR lentiviral vector drives on-target and off-target antigen modulation in leukemia cell lines. J Immunother Cancer.

[305] Grada Z, Hegde M, Byrd T, Shaffer DR, Ghazi A, Brawley VS, et al. TanCAR: a novel bispecific chimeric antigen receptor for cancer immunotherapy. Mol Ther - Nucleic Acids. 2013;2:e105. 10.1038/mtna.2013.32. 10.1038/mtna.2013.32 PMC3731887 23839099

[306] Roybal KT, Williams JZ, Morsut L, Rupp LJ, Kolinko I, Choe JH (2016). Engineering T cells with customized therapeutic response programs using synthetic notch receptors. Cell.

[307] Sadelain M (2014). Antigen-specific inhibitory chimeric antigen receptors (iCARs) as a self-regulating safety switch to constrain T cell–based therapies. Sci Exch.

[308] Zhang E, Xu H (2017). A new insight in chimeric antigen receptor-engineered T cells for cancer immunotherapy. J Hematol Oncol.

[309] Hofmann O, Caballero OL, Stevenson BJ, Chen Y-T, Cohen T, Chua R (2008). Genome-wide analysis of cancer/testis gene expression. Proc Natl Acad Sci.

[310] Sahin U, Türeci O, Chen YT, Seitz G, Villena-Heinsen C, Old LJ (1998). Expression of multiple cancer/testis (CT) antigens in breast cancer and melanoma: basis for polyvalent CT vaccine strategies. Int J Cancer.

[311] NR dos S, Torensma R, TJ de V, MW S DR, de B, Kater-Baats E (2000). Heterogeneous expression of the SSX cancer/testis antigens in human melanoma. Mod Pathol.

[312] Salmaninejad A, Zamani MR, Pourvahedi M, Golchehre Z, Hosseini Bereshneh A, Rezaei N (2016). Cancer/testis antigens: expression, regulation, tumor invasion, and use in immunotherapy of cancers. Immunol Investig.

[313] Chen Y-T, Hsu M, Lee P, Shin SJ, Mhawech-Fauceglia P, Odunsi K (2009). Cancer/testis antigen CT45: analysis of mRNA and protein expression in human cancer. Int J Cancer.

[314] Zendman AJW, de Wit NJW, van Kraats AA, Weidle UH, Ruiter DJ, van Muijen GNP (2001). Expression profile of genes coding for melanoma differentiation antigens and cancer/testis antigens in metastatic lesions of human cutaneous melanoma. Melanoma Res.

[315] Gjerstorff MF, Johansen LE, Nielsen O, Kock K, Ditzel HJ (2006). Restriction of GAGE protein expression to subpopulations of cancer cells is independent of genotype and may limit the use of GAGE proteins as targets for cancer immunotherapy. Br J Cancer.

[316] Greve KBV, Pøhl M, Olsen KE, Nielsen O, Ditzel HJ, Gjerstorff MF (2014). SSX2-4 expression in early-stage non-small cell lung cancer. Tissue Antigens.

[317] Obenaus M, Leitão C, Leisegang M, Chen X, Gavvovidis I, van der Bruggen P (2015). Identification of human T-cell receptors with optimal affinity to cancer antigens using antigen-negative humanized mice. Nat Biotechnol.

[318] Magee MS, Kraft CL, Abraham TS, Baybutt TR, Marszalowicz GP, Li P, et al. GUCY2C-directed CAR-T cells oppose colorectal cancer metastases without autoimmunity. Oncoimmunology. 2016;5:1–10. Taylor & Francis. Available from: 10.1080/2162402X.2016.1227897. 10.1080/2162402X.2016.1227897 PMC5087292 27853651

[319] Snook AE, Eisenlohr LC, Rothstein JL, Waldman SA (2007). Cancer mucosa antigens as a novel immunotherapeutic class of tumor-associated antigen. Clin Pharmacol Ther.

[320] Frick GS, Pitari GM, Weinberg DS, Hyslop T, Schulz S, Waldman SA (2005). Guanylyl cyclase C: a molecular marker for staging and postoperative surveillance of patients with colorectal cancer. Expert Rev Mol Diagn.

[321] Carrithers SL, Barber MT, Biswas S, Parkinson SJ, Park PK, Goldstein SD (1996). Guanylyl cyclase C is a selective marker for metastatic colorectal tumors in human extraintestinal tissues. Proc Natl Acad Sci U S A.

[322] Birbe R, Palazzo JP, Walters R, Weinberg D, Schulz S, Waldman SA (2005). Guanylyl cyclase C is a marker of intestinal metaplasia, dysplasia, and adenocarcinoma of the gastrointestinal tract. Hum Pathol.

[323] Schulz S, Hyslop T, Haaf J, Bonaccorso C, Nielsen K, Witek ME (2006). A validated quantitative assay to detect occult micrometastases by reverse transcriptase-polymerase chain reaction of guanylyl cyclase C in patients with colorectal cancer. Clin Cancer Res.

[324] Adam E. Snook, Michael S. Magee, Stephanie Schulz, and Scott A. Waldman. Self-tolerance eliminates CD4+ T, but not CD8+T or B, cells corrupting cancer immunotherapy. Eur J Immunol. 2014;44(7):1956–66. 10.1002/eji.201444539 PMC4107120 24771148

[325] Snook AE, Li P, Stafford BJ, Faul EJ, Huang L, Birbe RC (2009). Lineage-specific T-cell responses to Cancer mucosa antigen oppose systemic metastases without mucosal inflammatory disease. Cancer Res.

[326] Ponnusamy MP, Venkatraman G, Singh AP, Chauhan SC, Johansson SL, Jain M (2007). Expression of TAG-72 in ovarian cancer and its correlation with tumor stage and patient prognosis. Cancer Lett.

[327] Xu M, Real FX, Welt S, Schüssler MH, Oettgen HF, Old LJ (1989). Expression of TAG-72 in normal colon, transitional mucosa, and colon cancer. Int J Cancer.

[328] Pizzi C, Sgambato A, De Laurentiis M, Limite G, Panico L, Pettinato G (1999). TAG-72 expression and clinical outcome in primary breast cancer. Oncol Rep.

[329] Lottich SC, Johnston WW, Szpak CA, Delong ER, Thor A, Schlom J (1985). Tumor-associated antigen TAG-72: correlation of expression in primary and metastatic breast carcinoma lesions. Breast Cancer Res Treat.

[330] Murray JL, Macey DJ, Grant EJ, Rosenblum MG, Kasi LP, Zhang HZ (1995). Enhanced TAG-72 expression and tumor uptake of radiolabeled monoclonal antibody CC49 in metastatic breast cancer patients following alpha-interferon treatment. Cancer Res.

[331] Brenner PC, Rettig WJ, Sanz-Moncasi MP, Reuter V, Aprikian A, Old LJ (1995). TAG-72 expression in primary, metastatic and hormonally treated prostate cancer as defined by monoclonal antibody CC49. J Urol.

[332] Karan D, Johansson SL, Lin MF, Batra SK (2001). Expression of tumor-associated glycoprotein-72 (TAG-72) antigen in human prostatic adenocarcinomas. Oncol Rep.

[333] Osteen KG, Anderson TL, Schwartz K, Hargrove JT, Gorstein F (1992). Distribution of tumor-associated glycoprotein-72 (TAG-72) expression throughout the normal female reproductive tract. Int J Gynecol Pathol.

[334] Soisson AP, Berchuck A, Lessey BA, Soper JT, Clarke-Pearson DL, McCarty KS (1989). Immunohistochemical expression of TAG-72 in normal and malignant endometrium: correlation of antigen expression with estrogen receptor and progesterone receptor levels. Am J Obstet Gynecol.

[335] Stout JT, Caskey CT (1985). Hprt: gene structure, expression, and mutation.

[336] Melton DW, Ketchen AM, J Selfridge. Stability of HPRT marker gene expression at different gene-targeted loci: observing and overcoming a position effect. Nucleic Acids Res. 1997;25(19):3937–43. 10.1093/nar/25.19.3937 PMC146987 9380520

[337] Townsend MH, Felsted AM, Ence ZE, Piccolo SR, Robison RA, O’Neill KL (2017). Elevated expression of hypoxanthine guanine Phosphoribosyltransferase within malignant tissue. Cancer Clin Oncol.

[338] Townsend MH, Robison RA, O’Neill KL. A review of HPRT and its emerging role in cancer. Med Oncol. 2018;35:89. Springer US. Cited 22 May 2018. Available from: http://link.springer.com/10.1007/s12032-018-1144-1, https://www.ncbi.nlm.nih.gov/pubmed/28408844. 10.1007/s12032-018-1144-1 29730818

[339] Townsend MH, Anderson MD, Weagel EG, Velazquez EJ, Weber KS, Robison RA (2017). Non-small-cell lung cancer cell lines A549 and NCI-H460 express hypoxanthine guanine phosphoribosyltransferase on the plasma membrane. Onco Targets Ther.

[340] Weagel EG, Townsend MH, Anderson MD, Velazquez EJ, Weber KS, Robison RA (2017). Abstract 2149: unusual expression of HPRT on the surface of the colorectal cancer cell lines HT29 and SW620. Cancer Res.

[341] He Q, Zou L, Zhang PA, Lui JX, Skog S, Fornander T (2000). The clinical significance of thymidine kinase 1 measurement in serum of breast cancer patients using anti-TK1 antibody. Int J Biol Markers.

[342] Jagarlamudi KK, Hansson LO, Eriksson S. Breast and prostate cancer patients differ significantly in their serum thymidine kinase 1 ( TK1 ) specific activities compared with those hematological malignancies and blood donors : implications of using serum TK1 as a biomarker. 2015;1–13. 10.1186/s12885-015-1073-8 PMC4336758 25881026

[343] Li HX, Lei DS, Wang XQ, Skog S, He Q (2005). Serum thymidine kinase 1 is a prognostic and monitoring factor in patients with non-small cell lung cancer. Oncol Rep.

[344] Carlsson L, Larsson A, Lindman H (2009). Elevated levels of thymidine kinase 1 peptide in serum from patients with breast cancer. Ups J Med Sci.

[345] Weagel EG, Meng W, Townsend MH, Velazquez EJ, Brog RA, Boyer MW (2017). Biomarker analysis and clinical relevance of thymidine kinase 1 on the cell membrane of Burkitt’s lymphoma and acute lymphoblastic leukemia. Onco. Targets. Ther..

